# Inflammation‐associated intramyocellular lipid alterations in human pancreatic cancer cachexia

**DOI:** 10.1002/jcsm.13474

**Published:** 2024-05-09

**Authors:** Min Deng, Jianhua Cao, Gregory van der Kroft, David P.J. van Dijk, Merel R. Aberle, Andrej Grgic, Ulf P. Neumann, Georg Wiltberger, Benjamin Balluff, Frank G. Schaap, Ron M.A. Heeren, Steven W.M. Olde Damink, Sander S. Rensen

**Affiliations:** ^1^ Department of Surgery, NUTRIM School of Nutrition and Translational Research in Metabolism Maastricht University Maastricht The Netherlands; ^2^ Division of Imaging Mass Spectrometry, Maastricht Multimodal Molecular Imaging (M4i) Institute Maastricht University Maastricht The Netherlands; ^3^ Department of General, Visceral and Transplantation Surgery RWTH University Hospital Aachen Aachen Germany

**Keywords:** Cancer cachexia, Ceramides, Intramyocellular lipid, LC–MS/MS, Lipidomics, Mass spectrometry imaging, Muscle atrophy

## Abstract

**Background:**

Cancer cachexia is a multifactorial metabolic syndrome characterized by systemic inflammation and ongoing skeletal muscle loss resulting in weakness, poor quality of life, and decreased survival. Whereas lipid accumulation in skeletal muscle is associated with cancer cachexia as well as the prognosis of cancer patients, surprisingly little is known about the nature of the lipids that accumulate in the muscle during cachexia, and whether this is related to inflammation. We aimed to identify the types and distributions of intramyocellular lipids in patients with and without cancer cachexia.

**Methods:**

Rectus abdominis muscle biopsies were collected during surgery of patients with pancreatic ductal adenocarcinoma (*n* = 10 without cachexia, *n* = 20 cachectic without inflammation (CRP < 10 mg/L), *n* = 10 cachectic with inflammation (CRP ≥ 10 mg/L). L3‐CT scans were analysed to assess body composition based on validated thresholds in Hounsfield units (HU). Muscle sections were stained with Oil‐Red O and H&E to assess general lipid accumulation and atrophy. Untargeted lipidomic analyses were performed on laser‐microdissected myotubes using LC–MS/MS. The spatial distribution of intramyocellular lipids with differential abundance between groups was visualized by mass‐spectrometry imaging. Genes coding for inflammation markers and enzymes involved in *de novo* ceramide synthesis were studied by qPCR.

**Results:**

Muscle radiation attenuation was lower in cachectic patients with inflammation (median 24.3 [18.6–30.8] HU) as compared with those without inflammation (34.2 [29.3–38.7] HU, *P* = 0.033) or no cachexia (37.4 [33.9–42.9] HU, *P* = 0.012). Accordingly, intramyocellular lipid content was lower in non‐cachectic patients (1.9 [1.6–2.1]%) as compared with those with cachexia with inflammation (5.5 [4.5–7.3]%, *P* = 0.002) or without inflammation (4.8 [2.6–6.0]%, *P* = 0.017). Intramyocellular lipid accumulation was associated with both local IL‐6 mRNA levels (*r*_*s*_ = 0.57, *P* = 0.015) and systemic CRP levels (*r*_*s*_ = 0.49, *P* = 0.024). Compared with non‐cachectic subjects, cachectic patients had a higher relative abundance of intramyocellular glycerophospholipids and a lower relative abundance of glycerolipids. Furthermore, increases in several intramyocellular lipids such as SM(d36:1), PC(34:1), and TG(48:1) were found in cachectic patients with inflammation and correlated with specific cachexia features. Altered intramyocellular lipid species such as PC(34:1), LPC(18:2), and TG(48:1) showed an uneven distribution in muscle sections of cachectic and non‐cachectic patients, with areas featuring abundance of these lipids next to areas almost devoid of them.

**Conclusions:**

Intramyocellular lipid accumulation in patients with cachexia is associated with both local and systemic inflammation, and characterized by changes in defined lipid species such as glycerolipids and glycerophospholipids.

## Introduction

Cancer cachexia is a multifactorial wasting syndrome characterized by the involuntary loss of body weight and skeletal muscle.^1^ This complex syndrome is associated with poor prognosis and leads to poor quality of life, resistance to anti‐cancer treatment, and cancer‐related morbidity and mortality.^1^ Cancer cachexia affects up to 80% of patients with advanced cancer and accounts for 20% of cancer‐related deaths.^2^ The incidence and prevalence of cachexia varies depending on tumour type and stage. It is particularly common in patients with pancreatic and gastro‐oesophageal cancers, followed by head–neck and lung cancers. Fat infiltration in skeletal muscle is frequently referred to as myosteatosis. It is defined by the ectopic accumulation of fat in skeletal muscle which may occur as intermuscular adipose tissue, intramuscular adipose tissue, as well as intramyocellular lipids. Although myosteatosis is often associated with sarcopenia (low skeletal muscle mass), it represents a different aspect of skeletal muscle status and is thought to reflect muscle quality. Myosteatosis has been associated with insulin resistance, poor physical condition, higher mortality, and shorter disease‐free survival in cancer patients.^3^, ^4^ For example, Aleixo and colleagues showed that cancer patients with myoteatosis had 75% greater mortality risk compared with those without myosteatosis.^5^

Lipids are hydrophobic molecules including glycerolipids, glycerophospholipids, sterol lipids, and sphingolipids.^6^ They represent a diverse group of biomolecules with various functions that vary depending on their structure, and are commonly composed of two distinct types of biochemical building blocks: fatty acids and cholesterol. Lipids not only serve as essential components of cell membranes, but are also involved in metabolism and cell signalling.^7^ It is widely recognized that ectopic lipid accumulation is associated with type 2 diabetes as well as cardiovascular diseases.^8^ Furthermore, altered lipid metabolism can regulate a variety of carcinogenic processes including tumour proliferation, migration, invasion, and metastasis.^9^, ^10^ Overexpression of fatty acid synthase (FASN), a key enzyme involved in lipid metabolism, is found in most solid tumours, just like other lipogenic enzymes such as ATP‐citrate lyase (ACLY) and acetyl‐CoA carboxylase alpha (ACACA).^11^ Moreover, increased cholesterol, phospholipids, and sphingolipid concentrations have been observed in malignant tumours.^12^, ^13^

Sphingolipids are a class of lipids that have critical roles in the regulation of several biological processes like cell growth, differentiation, and apoptosis. Sphingolipid metabolites, such as ceramides, have been implicated in the progression of cachexia. For example, elevated plasma sphingolipids such as SM(16:0), SM(24:1), CER(16:0), CER(24:1), HCER(16:0), and HCER(24:1) have been observed in mice and humans with cancer cachexia, and these ceramides may contribute to muscle wasting.^14^ Alterations in circulating glycerophospholipids, sphingolipids, and free fatty acids have also been reported in cancer patients with cachexia and were associated with body weight loss as well as levels of pro‐inflammatory cytokines.^15^, ^16^, ^17^ Moreover, the number and size of intramyocellular lipid droplets increase with the progression of cancer cachexia.^18^ To date, little is known about the nature of the lipids that accumulate in skeletal muscle during cancer cachexia, and whether they are correlated to the reported alterations in plasma levels of specific lipids and/or inflammatory features.

Liquid chromatography–tandem mass spectrometry (LC–MS/MS)‐based lipidomics is a high‐throughput analytical technology that permits simultaneous detection and quantification of hundreds of lipids. Several studies have applied LC–MS/MS approaches to study metabolite, lipid and protein profiles in plasma of cachectic cancer patients.^14^, ^15^ More recently, Kunzke and colleagues utilized matrix‐assisted laser desorption/ionization‐mass spectrometry imaging (MALDI‐MSI) to examine amino acid alterations in skeletal muscle from cachectic and non‐cachectic mice and patients.^19^ MALDI‐MSI is a powerful technique for the *in situ* analysis of many biomolecules with high sensitivity. As a label‐free technique, MALDI‐MSI has been widely used in clinical studies to determine the spatial distribution of new drugs and to discover new predictive and prognostic biomarkers in cancer research.^20^, ^21^

In this study, we sought to deepen our knowledge of lipid metabolism in the skeletal muscle of patients with cancer cachexia by using a LC–MS/MS‐based approach. Our primary aim was to investigate intramyocellular lipid alterations in cancer cachexia in relation to the inflammatory status of these patients. Our secondary aim was to visualize the spatial distribution of intramyocellular lipids by MALDI‐MSI. We found that intramyocellular lipid accumulation in patients with cachexia features a higher relative abundance of glycerophospholipids, and a lower relative abundance of glycerolipids. Furthermore, intramyocellular lipid accumulation was positively correlated with skeletal muscle IL‐6 expression and systemic CRP levels. In addition, certain intramyocellular lipids were specifically elevated in the muscle of patients with cancer cachexia. To the best of our knowledge, this is the first work to investigate the composition and distribution of intramyocellular lipids in patients with cancer cachexia.

## Methods

### Patient inclusion and sample collection

Forty‐eight patients with pancreatic ductal adenocarcinoma (PDAC) undergoing pancreaticoduodenectomy at the Maastricht University Medical Centre (MUMC+) or the Uniklinikum Aachen were enrolled between 2014–2021. Rectus abdominis muscle samples were obtained at abdominal incision during surgery from 40 out of 48 patients and immediately snap‐frozen in liquid nitrogen and kept at −80°C until further analysis. The study cohort was expanded to include the 8 patients who lacked muscle biopsy data but had undergone computed tomography (CT) imaging. These CT scans were leveraged to perform body composition analyses. This study was approved by both the Medical Ethical Committee at Maastricht University (METC) and the Medical Ethical Committee at RWTH‐Aachen. All patients provided written informed consent for tissue biopsy.

### Computed tomography‐based body composition

Pre‐operative CT scans were performed with a spiral CT scanner for diagnostic purposes. CT scans were analysed using SliceOmatic 5.0 software (TomoVision, Magog, Canada). Using pre‐established validated thresholds of Hounsfield units (HU), the total cross‐sectional areas (cm^2^) of skeletal muscle tissue (−29 to 150 HU), visceral adipose tissue (SAT, −150 to −50 HU), and subcutaneous tissue (VAT, −150 to −50 HU) at the third lumbar vertebra (L3) were evaluated. In addition, the skeletal muscle radiation attenuation (SMRA) was assessed by calculating the average HU value of the total muscle area within the specified range of −29 to 150 HU. The total areas of skeletal muscle, visceral adipose tissue and subcutaneous tissue were normalized for stature (m^2^) and expressed as skeletal muscle index (L3‐SMI), L3‐VAT index, and L3‐SAT index in cm^2^/m^2^. Sarcopenia was defined using established cut‐offs in patients with pancreatic cancer as L3‐SMI < 45.1 cm^2^/m^2^ in males and <36.9 cm^2^/m^2^ in females.^22^

### Cryosection and sample preparation for mass spectrometry imaging

Frozen rectus abdominis muscle tissue samples were cryosectioned at 12 μm thickness using a cryo‐microtome (Leica, Rijswijk, The Netherlands) at −20°C and thaw mounted onto either indium tin oxide (ITO) coated conductive slides (CG‐40IN‐S115, Delta Technologies, Loveland, CO, USA) or normal glass slides (VWR International bvba, Belgium). ITO slides were used for MSI experiments and afterwards stained with H&E. Oil Red O staining was performed on normal glass slides. At least 3 sections were obtained from each sample for MSI, H&E staining, and Oil Red O staining. All slides were stored at −80°C until further processing.

All ITO glass slides were dried in a vacuum desiccator for 15 min and fiducial markers (Tipp‐Ex, BIC, France) were applied to the ITO slide before matrix deposition. A 15 mg/mL 2,5‐dihydroxybenzoic acid (DHB, Sigma Aldrich, St. Louis, MO, USA) matrix solution was prepared in 2:1 (vol:vol) chloroform: methanol and homogeneously deposited onto the slides using an automated, temperature‐controlled spraying system (TM‐sprayer, HTX Technologies, Chapel Hill, NC, USA.). Briefly, 10 layers were sprayed at 50°C with a constant flow rate of 0.12 mL/min, a track spacing at 3 mm and at a speed of 1200 mm/min combined with 30 s drying time between each layer. Samples were analysed immediately after matrix application.

### Oil Red O staining and haematoxylin‐eosin staining

Intramyocellular lipids in skeletal muscle sections of the first consecutive 21 patients (Table S1) that were included were visualized by Oil Red O (ORO) staining. Briefly, frozen sections were equilibrated for 30 min at room temperature and subsequently fixed in 10% neutral buffered formalin for 10 min. Thereafter, a quick wash with 60% isopropanol was performed, and slides were incubated in 60% ORO [(dissolved in 100% isopropanol)/MilliQ: 3/2 (vol/vol)] solution for 15 min at room temperature. Slides were rinsed in H_2_O for 10 s and covered with a coverslip using aqueous mounting gel (Dako Faramount Aqueous Mounting Medium, USA). Images of the stained muscle sections were captured using an Aperio CS2 digital pathology slide scanner (Leica Biosystems, Wetzlar, Germany), and analysed using ImageJ software (National Institutes of Health). To quantify intramyocellular lipid content, 15 myofibers from each slide were randomly selected, with exclusion of the myofiber membrane. The percentage of area occupied by intramyocellular lipids ((total area occupied by intramyocellular lipids/total area of myofibers)*100) was calculated.

Haematoxylin and eosin (H&E) staining was performed after MALDI‐MSI to allow alignment of molecular and histological information. Matrix was removed by immersion in 70% ethanol for 3 min. A standard H&E protocol was then used. High‐resolution optical images of stained tissues were obtained using an Aperio CS2 digital pathology slide scanner (Leica Biosystems, Wetzlar, Germany). The minimal Feret's diameter of the myofibers was calculated using ImageJ software. All experiments and analyses were done by one investigator who was blinded for the study groups.

### Sample preparation for lipidomics

To prevent confounding by intermuscular adipocytes, laser capture microdissection (Leica LMD7000, Leica Microsystems, Wetzlar, Germany) was applied to isolate myotubes from muscle tissue sections for later lipid extraction. Laser capture microdissection was performed using the following parameters: wavelength = 349 nm, power = 44, aperture = 15, speed = 10, specimen balance = 15, head current = 100%, and pulse frequency = 119 Hz. Microdissected muscle cells (around 2.5 mm^2^) were directly collected in an empty Eppendorf tube. Lipid extraction was performed using the protocol described by Breitkopf^23^ with minor modifications.

### Untargeted lipidomics analysis

LC–MS/MS‐based lipidomics was conducted with a Thermo Scientific™ Vanquish™ Ultra‐High‐Performance LC system interfaced to an Orbitrap Exploris™ 480 Mass Spectrometer (Thermo Fisher, Bremen, Germany). Briefly, 5 μL of every lipid extract was injected into a Thermo Scientific™ Accucore™ HILIC HPLC column (100 × 2.1 mm, 2.6 μm, Thermo Scientific) using the same LC method as described by Breitkopf et al.^23^

The LC–MS/MS‐based lipidomics spectrometer was operated in the positive ionization mode using the same approach as described by Breitkopf *et al* ^23^: sheath gas flow rate: 35 arb, auxiliary gas flow rate: 12, sweep gas flow rate: 3, spray voltage: 4350 V, ion transfer tube temperature: 330°C, vaporizer temperature: 325°C, RF lens level: 55, auxiliary gas heater: 435°C, mass range: *m/z* 200–1450, mass resolution: 60 000 (at *m/z* 200), HCD collision energy: 30. Raw peak areas were normalized to the area of microdissected muscle cells. LipidSearch software v.5.0 was used to identify lipid species. The following settings were used for lipid search and lipid alignment. For lipid search: Precursor mass tolerance: 5 ppm; Product mass tolerance: 10 ppm; Product intensity threshold: 1.0%; Product threshold type: relative; Main isomer peak filter: ON; ID Quality filter: A, B, C; Adducts (pos. ion): [M + H]^+^, [M + Na]^+^, [M + NH_4_]^+^, and [M + H ‐ H_2_O]^+^. For lipid alignment: retention time tolerance: 0.05 min; all isomer peak filter: ON; ID Quality filter: A, B, C.

### Matrix‐assisted laser desorption/ionization‐mass spectrometry imaging analyses

MSI analyses of muscle cryosections of nine randomly selected patients [no cachexia (*n* = 3), cachexia without inflammation (*n* = 3), cachexia with inflammation (*n* = 3), see Table S2 for clinical patient information] were performed with a hybrid‐source system (timsTOF fleX, Bruker Daltonik, Bremen, Germany) in positive ionization mode at 20*20 μm^2^ spatial resolution within a mass range of *m/z* 300–1500. The laser operated at a frequency of 10 000 Hz and 150 shots per pixel. timsControl 2.0 and FlexImaging 5.0 (Bruker Daltonik, Bremen, Germany) were used for data acquisition. The MSI data from TIMS‐TOF were imported into SCilS Lab 2022a (SCiLS GmbH, Bremen, Germany) and coregistered with the histological images. The peak interval width was set to *m/z* 0.02. Root mean square values were used for normalization.

Additionally, high mass resolution (240 000 full‐width at half‐maximum at *m/z* 200) MSI experiments were performed with an Orbitrap Elite Mass Spectrometer (Thermo Fisher, Bremen, Germany) equipped with an ion‐funnel‐based MALDI interface (Spectroglyph LLC, Kennewick, WA, USA). Briefly, tissue sections were analysed in the positive ionization mode within the mass range of *m/z* 300–1500 using the data‐dependent acquisition (DDA)‐imaging method described by Ellis et al.^24^ The DDA experiment was conducted with the following parameters: stage step size = 25*50 μm^2^; ion injection time = 50 ms; normalized collision energy = 30 eV; and activation q = 0.17.

All DDA data were imported in LipostarMSI,^25^ and lipid identification was performed with precursor *m/z* tolerance at 0.00 ± 3 ppm and MS/MS *m/z* tolerance at 0.25 amu ± 0 ppm.

### RNA isolation and qRT‐PCR

Total RNA was isolated from frozen skeletal muscle using TRI Reagent (Sigma, St. Louis, MO, USA) according to the manufacturer's protocol. RNA concentration and purity were quantified with a DeNovix DS‐11 spectrophotometer. cDNA was synthesized using a total of 750 ng RNA by the SensiFAST cDNA Synthesis Kit according to the manufacturer's instructions (Bioline GmbH, Germany). To quantify mRNA expression levels, RT‐qPCR was performed according to previously published protocols. Relative mRNA levels were calculated using LinRegPCR (Version 2016.1) and normalized to the reference gene ribosomal phosphoprotein P0 (RPLP0/36B4). Primer sequences are provided in Table S3.

### Statistical analysis

Statistical analysis was performed using Prism 7.0 for Windows (GraphPad Software Inc., San Diego, CA), and R (R‐4.2.0 for Windows). Unless otherwise indicated, data are presented as median with interquartile range (IQR). Differences between groups were analysed by the Kruskal–Wallis test followed by Dunn's post‐testing for non‐parametric variables. Unpaired Student's *t*‐test was used for parametric variables. *P*‐values <0.05 were considered statistically significant. Spearman's correlation matrix was generated by the Corrplot R package.^26^ Lipid data were uploaded into MetaboAnalyst 5.0^27^ for normalization [log transformation (base 10)] and partial least squares discriminant analysis (PLS‐DA).

## Results

### Cachexia related characteristics of the study cohort

In this study, a total of 48 patients with PDAC were included. According to the international consensus definition of cancer cachexia,^28^ the cohort was divided into non‐cachectic and cachectic groups. Given that systemic inflammation plays a vital role in cancer‐associated cachexia as well as in insulin resistance‐related lipid accumulation in muscle,^1^, ^29^ cachectic patients were subdivided into two subgroups: cachexia with inflammation and cachexia without inflammation, using a cut‐off value of C‐reactive protein (CRP) of 10 mg/L. The patient's characteristics are shown in Table 1. The study cohort consisted of 18 (37.5%) females and 30 males (62.5%) with a median age of 69.5 years and a median body mass index of 23.0 kg/m^2^. CRP levels of cachectic patients with inflammation were 22.6 [13.9–36.3] mg/L. CRP levels of both cachectic patients without inflammation and patients without cachexia were significantly lower (3.8 [1.1–5.2] mg/L, *P* < 0.001, and 4.4 [1.2–8.3] mg/L, *P* = 0.001, respectively). The median body weight loss percentage of the non cachexia group was 1.6 [0.0–2.6]%, which was significantly lower than in cachectic patients without inflammation (13.0 [9.9–16.4]%, *P* < 0.001) or with inflammation (8.7 [8.0–11.0]%, *P* = 0.003).

**Table 1:** Basic characteristics of patients within the study cohort

	Overall	No cachexia	Cachexia	Cachexia	*P*
			Without inflammation	With inflammation	
*n*	48	12	24	12	
Age (years)	69.5 (59.8, 75.2)	59.5 (55.5, 71.0)	70.0 (60.8, 75.2)	73.0 (68.8, 76.0)	0.094
Sex = F/M (%)	18/30 (37.5/62.5)	6/6 (50.0/50.0)	7/17 (29.2/70.8)	5/7 (41.7/58.3)	0.398
BMI (kg/m^2^)	23.0 (21.9, 25.8)	24.0 (22.4, 26.6)	22.9 (21.8, 25.9)	22.5 (21.2, 24.5)	0.464
Weight loss (%)	8.8 (4.7, 14.2)	1.6 (0.0, 2.6)	13.0 (9.9, 16.4)^a^	8.7 (8.0, 11.0)^a^	<0.001
Handgrip strength (kg)	30.0 (23.8, 38.5)	30.0 (25.2, 40.0)	32.0 (26.0, 40.0)	24.0 (21.5, 32.0)	0.226
SMRA (HU)	33.4 (27.6, 38.6)	37.4 (33.9, 42.9)	34.2 (29.3, 38.7)	24.3 (18.6, 30.8)^a^, ^b^	0.009
L3‐SMI (cm^2^/m^2^)	40.5 (36.7, 45.7)	38.7 (32.7, 43.4)	43.5 (38.9, 47.5)	38.6 (34.5, 45.6)	0.171
Male	44.2 (39.7, 50.3)	44.2 (41.6, 45.8)	45.1 (40.8, 50.6)	39.0 (37.4, 47.2)	0.493
Female	36.0 (31.3, 39.1)	32.5 (30.8, 35.7)	39.1 (36.7, 40.4)	34.8 (29.2, 38.9)	0.117
L3‐VATI (cm^2^/m^2^)	33.0 (19.2, 74.1)	24.8 (18.2, 47.3)	32.5 (15.2, 86.8)	44.5 (28.1, 65.0)	0.390
Male	51.5 (25.7, 88.3)	49.8 (29.9, 81.8)	58.6 (20.3, 87.5)	48.4 (37.0, 77.8)	0.894
Female	22.6 (13.9, 32.9)	19.6 (17.3, 23.6)	15.9 (10.2, 29.2)	40.4 (28.9, 41.0)	0.226
L3‐SATI (cm^2^/m^2^)	45.0 (38.6, 63.8)	46.2 (36.4, 72.4)	43.6 (36.4, 53.0)	45.9 (42.7, 63.7)	0.606
Male	43.1 (32.9, 55.8)	45.0 (32.5, 65.6)	40.7 (31.1, 48.9)	46.2 (41.7, 62.5)	0.559
Female	46.8 (42.3, 76.8)	46.2 (39.7, 77.4)	48.8 (43.7, 74.7)	45.5 (43.6, 62.5)	0.972
CRP (mg/L)	5.0 (1.4, 11.2)	4.4 (1.2, 8.3)	3.8 (1.1, 5.2)	22.6 (13.9, 36.3)^a^, ^b^	<0.001
Albumin (g/dL)	4.0 (3.5, 4.4)	4.3 (4.2, 4.4)	3.9 (3.4, 4.4)	3.8 (3.1, 4.3)	0.090
CRP/albumin ratio	1.2 (0.3, 3.2)	1.0 (0.2, 1.5)	0.9 (0.3, 1.9)	6.6 (3.8, 8.9)^a^, ^b^	<0.001
Cancer stage (%)					0.150
IA	2 (4.3)	0 (0.0)	1 (4.3)	1 (8.3)	
IB	1 (2.1)	0 (0.0)	0 (0.0)	1 (8.3)	
IIA	5 (10.6)	1 (8.3)	3 (13.0)	1 (8.3)	
IIB	21 (44.7)	5 (41.7)	7 (30.4)	9 (75.0)	
III	3 (6.4)	1 (8.3)	2 (8.7)	0 (0.0)	
IV^c^	6 (12.8)	1 (8.3)	5 (21.7)	0 (0.0)	
Unknown	9 (19.1)	4 (33.3)	5 (21.7)	0 (0.0)	
Neoadjuvant chemotherapy (%)					0.030
No	27 (56.2)	7 (58.3)	10 (41.7)	10 (83.3)	
Yes	12 (25.0)	5 (41.7)	6 (25.0)	1 (8.3)	
Unknown	9 (18.8)	0 (0.0)	8 (33.3)	1 (8.3)	

The data are presented as median + IQR. Groups were compared using the Kruskal–Wallis test followed by Dunn's post‐testing.

^a^ Significant difference in comparison to the no cachexia group.

^b^ Significant difference in comparison to the cachexia without inflammation group. BMI: body mass index; HU: Hounsfield unit; SMRA: skeletal muscle radiation attenuation; L3‐SMI: L3‐muscle index; L3‐VATI: L3‐visceral adipose tissue index; L3‐SATI: L3‐subcutaneous adipose tissue index; CRP: C‐reactive protein.

^c^ Patients underwent exploratory surgery, no resection.

**Figure 1:**
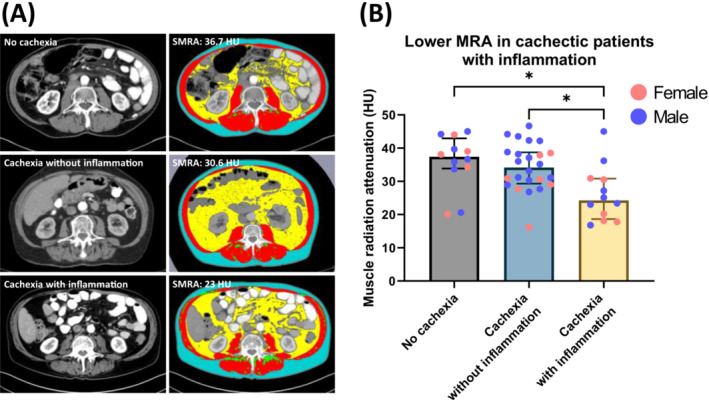
Comparison of body composition between non‐cachectic patients and cachectic patients with or without inflammation. (A) Original cross‐sectional CT images at the third lumbar vertebra from a non‐cachectic patient (panel A, top left), a cachectic patient without inflammation (panel A, middle left), and a cachectic patient with inflammation (panel A, bottom left), and CT segmentation using SliceOmatic for these respective patients, with SMRA values displayed. Colour‐coding: skeletal muscle (red), subcutaneous adipose tissue (blue), intermuscular adipose tissue (green), and visceral adipose tissue (yellow). (B) Comparison of SMRA in PDAC patients without cachexia (*n* = 12) and in cachectic patients with (*n* = 12) or without (*n* = 24) inflammation. Scatter plots show the median with interquartile range and individual data points in each group. For statistical analysis, the Kruskal–Wallis test followed by Dunn's multiple comparisons test was used. Significant differences among the groups are signified by asterisks (*P < 0.05).

CT scan‐based body composition analysis (Figure 1A) showed that 69% (33/48) of patients were sarcopenic, with a median L3‐SMI of 36.0 cm^2^/m^2^ for females and 44.2 cm^2^/m^2^ for males. The median L3‐VAT and L3‐SAT indices were 22.6 and 46.8 cm^2^/m^2^ for females and 51.5 and 43.1 cm^2^/m^2^ for males, respectively. Intriguingly, cachectic patients with systemic inflammation displayed a significantly lower SMRA than cachectic patients without inflammation and patients without cachexia (24.3 [18.6–30.8] HU vs. 34.2 [29.3–38.7] HU, *P* = 0.033 vs. 37.4 [33.9–42.9] HU, *P* = 0.012, Figure 1B), indicating increased muscle lipid content in patients with cachexia and inflammation.

Figure S1 shows a correlation matrix that illustrates the correlation between relevant patient characteristics and body composition parameters in the study cohort. As expected, SMRA was negatively correlated with age (*r*_*s*_ = −0.45, *P* = 0.001) and positively correlated with handgrip strength (*r*_*s*_ = 0.29, *P* = 0.045). Intriguingly, we also observed a negative correlation between SMRA and systemic CRP levels (*r*_*s*_ = −0.30, *P* = 0.035). Furthermore, SMRA reflecting lipid content within muscle fibres correlated inversely with the amount of visceral and subcutaneous adipose tissue (VATI, *r*_*s*_ = −0.42, *P* = 0.003, and SATI, *r*_*s*_ = −0.42, *P* = 0.003).

### Increased intramyocellular lipid accumulation in cachectic PDAC patients

**Figure 2:**
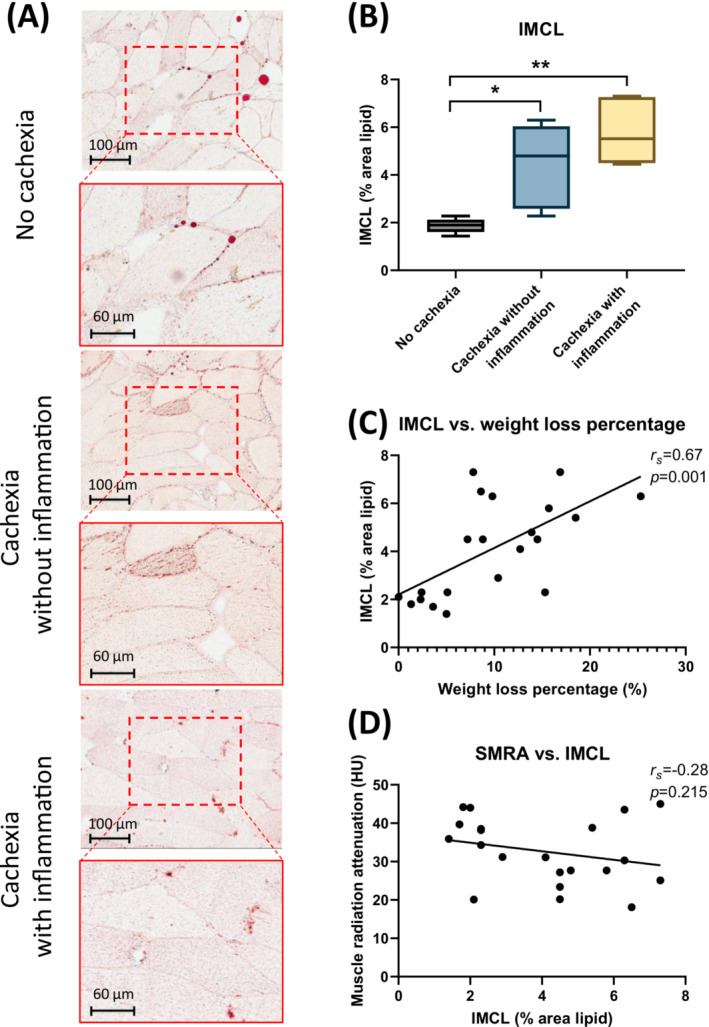
Intramyocellular lipid content and its relationship with body weight loss and CT‐derived muscle radiation attenuation in PDAC patients. (A) Representative images of Oil‐Red O stained abdominal muscle tissue from patients without cachexia (top panel), cachectic patients without inflammation (middle panel), and cachectic patients with inflammation (bottom panel). (B) Quantitation of intramyocellular lipid (IMCL) content (%) in muscle of non‐cachectic patients and cachectic patients with or without inflammation. (C) IMCL content (%) is positively correlated with body weight loss (%) but not correlated with skeletal muscle radiation attenuation (SMRA) *(D)* as assessed by L3‐CT‐scan analysis. No cachexia (*n* = 6); cachectic without inflammation (*n* = 9); cachectic with inflammation (*n* = 6). For statistical analysis, the Kruskal–Wallis test followed by Dunn's multiple comparisons test was used; significant differences among the groups are signified by asterisks (*P < 0.05, **P < 0.01). Spearman's rank correlation coefficient (*r*_*s*_) and level of significance are indicated in the respective plots.

To assess whether intramyocellular lipid accumulation was associated with cachexia, we performed Oil red O staining on skeletal muscle tissue sections of non‐cachectic PDAC patients and cachectic PDAC patients with or without inflammation (Figure 2A). The degree of lipid accumulation was significantly higher in cachectic patients with systemic inflammation as compared with non‐cachectic patients (5.5 [4.5–7.3]% vs. 1.9 [1.6–2.1]%, *P* = 0.002, Figure 2B). Similarly, lipid content of muscle was significantly higher in cachectic patients without inflammation than in non‐cachectic patients (4.8 [2.6–6.0]% vs. 1.9 [1.6–2.1]%, *P* = 0.017, Figure 2B). However, no significant difference in lipid accumulation was observed between cachectic patients with and without inflammation (5.5 [4.5–7.3]% vs. 4.8 [2.6–6.0]%, *P* = 0.900, Figure 2B). Furthermore, correlation analyses showed that intramyocellular lipid content positively correlated with body weight loss (*r*_*s*_ = 0.67, *P* = 0.001, Figure 2C). Intramyocellular lipid content was not correlated with SMRA (*r*_*s*_ = −0.28, *P* = 0.215, Figure 2D). Histological assessment of skeletal muscle sections of PDAC patients (see Table S4 for clinical information) showed a notable leftward shift in the frequency of smaller muscle fibres in cachectic PDAC patients, which was significant for the group with inflammation as compared with the non‐cachectic patients (Figure S2, *P* < 0.05).

### Intramyocellular lipid accumulation is associated with both local and systemic inflammation in PDAC patients

Inflammation is one of the key drivers of muscle wasting and ectopic fat accumulation in muscle.^29^ To evaluate local inflammation in skeletal muscle from PDAC patients with or without cachexia, we performed qRT‐PCR to examine mRNA expression of inflammatory cytokines tumour necrosis factor‐alpha (TNF‐α), interleukin‐6 (IL‐6), and interleukin‐1beta (IL‐1β). Although the mRNA expressions of TNF‐α and IL‐6 tended to be higher in skeletal muscle of cachectic patients as compared with non‐cachectic patients, the differences were not significant (Figure S3A (upper panel), S3B (upper panel), *P* = 0.055 and *P* = 0.080, respectively). Expression of TNF‐α and IL‐6 did not differ significantly between cachectic subgroups with and without inflammation [(Figure S3A (lower panel) and S3B (lower panel)]. IL‐1β was barely expressed in skeletal muscle of PDAC patients (data not shown). We next performed correlation analysis between intramyocellular lipid content in the skeletal muscle of PDAC patients and markers of local as well as systemic inflammation. As shown in Figures S3C–E, intramyocellular lipid content was not correlated with skeletal muscle TNF‐α mRNA (*r*_*s*_ = 0.32, *P* = 0.182), but significantly correlated with local IL‐6 mRNA (*r*_*s*_ = 0.57, *P* = 0.015) and systemic CRP levels (*r*_*s*_ = 0.49, *P* = 0.024).

### Altered intramyocellular glycerolipids and glycerophospholipids in PDAC patients with cachexia

**Figure 3:**
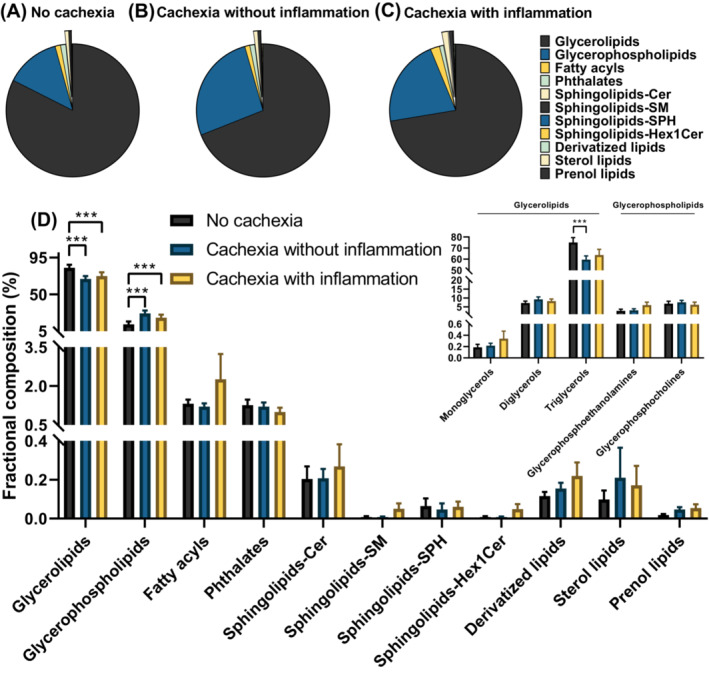
Distribution of intramyocellular lipid classes in relation to cachexia and systemic inflammation. Intramyocellular lipids in non‐cachectic PDAC patients (*n* = 10) (A), and cachectic PDAC patients with (*n* = 10) (B) or without (*n* = 20) (C) inflammation were analysed by LC–MS/MS. (D) Comparison of the relative quantities of major lipid classes between non‐cachectic patients and cachectic patients with or without inflammation. Statistical analyses were performed using multiple *t*‐tests with a false discovery rate (FDR) < 0.01. Data in the bar graph are presented as mean + SEM (***P < 0.001).

Alterations in specific lipid classes such as sphingolipids, triacylglycerols (TAG) and diacylglycerols (DAG) have been reported to be associated with muscle wasting in various pathologies including cancer.^30^ We performed untargeted lipidomics on myotubes isolated by laser capture microdissection to study intramyocellular lipid composition in 40 PDAC patients (Table S5) who underwent muscle biopsies, exploring its correlation with cachexia. In total, eight different lipid classes including 386 distinct lipid species were identified by LC–MS/MS (Table S6). The major lipid classes identified were glycerolipids (average 74.6% of all detected species, across all samples), glycerophospholipids (20.4%), sphingolipids (1.9%), and fatty acyls (1.6%). The fractional contribution of intramyocellular glycerolipids in the no cachexia group was 82.5 ± 3.9%, which was significantly higher than in cachectic patients without inflammation (69.0 ± 3.4%, *P* < 0.001) or with inflammation (72.4 ± 4.6%, *P* = 0.023) (Figure 3D). In contrast, intramyocellular glycerophospholipids fractional representation was significantly lower in patients without cachexia (13.3 ± 3.5%) than in cachectic patients without inflammation (26.6 ± 3.6%, *P* < 0.001) or cachectic patients with inflammation (21.4 ± 3.8%, *P* < 0.001) (Figure 3D). While no significant differences in intramyocellular fatty acyls and sphingolipids [ceramides (Cer), sphingomyelins (SM), and hexosyl ceramides (HexCer)] were observed, these lipid classes tended to be increased in cachectic PDAC patients with inflammation as compared with those patients without cachexia.

### Intramyocellular lipid alterations in PDAC patients in relation to cachexia and inflammation

**Figure 4:**
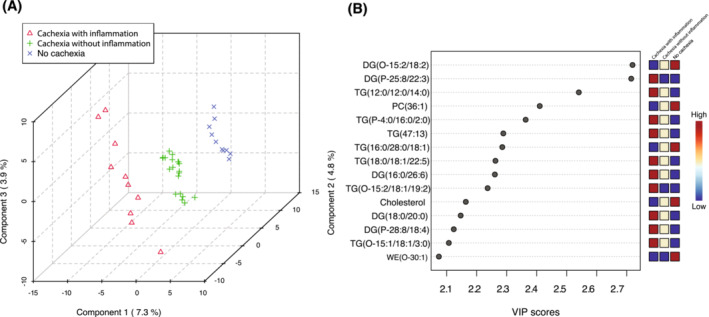
Partial least square discriminant analysis (PLS‐DA) models discriminate cachectic patients with inflammation (*n* = 10) from patients without inflammation (*n* = 20) and from non‐cachectic patients (*n* = 10). (A) PLS‐DA score plots showed clustering of patient groups. (B) VIP scores of top 15 lipid species identified by PLS‐DA in descending order of importance. The heatmaps on the right indicate the relative concentrations of the corresponding intramyocellular lipid species in each group under study.

Next, we applied a supervised model partial least squares discriminant analysis (PLS‐DA) to identify potential lipid markers that could discriminate between the three groups (no cachexia, cachexia with inflammation, and cachexia without inflammation). As shown in Figure 4A, the PLS‐DA score plot revealed a clear separation among the groups according to cachectic and inflammatory status. Variable importance in the projection (VIP) scores were used to estimate the importance of each lipid species in the PLS‐DA model. Only the variables with VIP > 2.0 were considered relevant for group discrimination. VIP scores of top 15 lipid species identified by PLS‐DA are shown in Figure 4B. The clustering (Figure 4A) was related to high levels of several glycerolipids [such as DG(P‐25:8/22:3), TG(12:0/12:0/14:0), and TG(P‐4:0/16:0/2:0)] observed in the muscle of patients with cachexia with inflammation compared with those without cachexia.

### No differences in intramyocellular ceramides between cachectic and non‐cachectic patients

**Figure 5:**
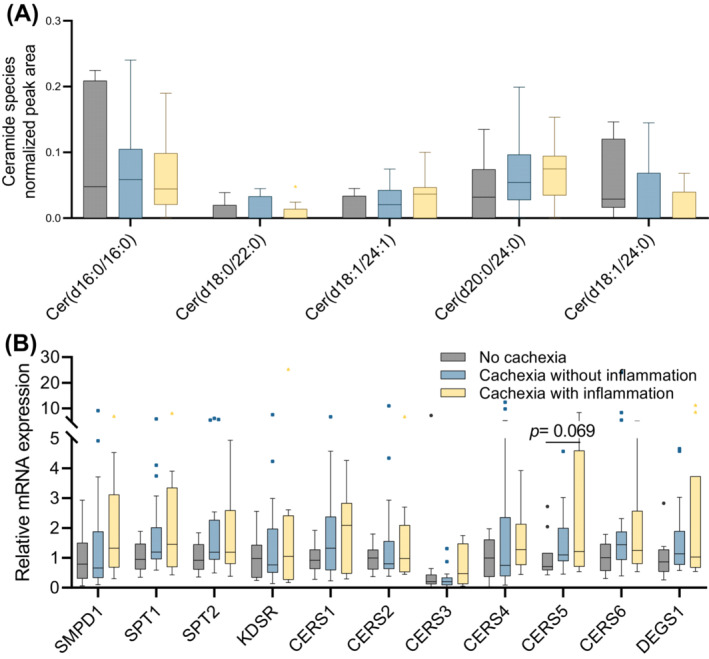
Ceramide content and expression of genes encoding enzymes controlling *de novo* ceramide synthesis in skeletal muscle of cachectic versus non‐cachectic PDAC patients. (A) Intramyocellular ceramide species in non‐cachectic patients (*n* = 10) and cachectic patients with (*n* = 10) or without inflammation (*n* = 20). (B) mRNA expression of genes coding for enzymes involved in *de novo* ceramides synthesis in skeletal muscle of PDAC patients. Statistical analyses were performed using multiple *t*‐tests with a false discovery rate (FDR) < 0.01 for Figure 7A, and ***P < 0.001 indicates differences from the control group (no cachexia). Statistical comparison for multiple groups in Figure 7B was evaluated using the Kruskal–Wallis test followed by Dunn's multiple comparisons test. The line reflects the median; the hinges of the boxes are drawn at the 25th and 75th percentile. The dots, squares and triangles in panels (A) and (B) reflect the outliers as defined by the Tukey method.

Given that elevated plasma ceramides have been proposed to be a feature of cancer cachexia in both mice and humans,^14^ and as intramyocellular ceramides fractional representation tended to be increased in cachectic patients with inflammation compared with cachectic patients without inflammation or without cachexia (Figure 3D), we next investigated whether the composition of specific intramyocellular ceramides was altered in cancer cachexia. Although ceramides Cer(d18:1/24:1) and Cer(d20:0/24:0) tended to be increased in cachectic patients with inflammation as compared with cachectic patients without inflammation or patients without cachexia, the differences were not significant (Figure 5A). In contrast, the abundance of intramyocellular Cer(d18:1/24:0) tended to be lower in cachectic patients with inflammation as compared with non‐cachectic patients (Figure 5A). Furthermore, no differences in mRNA expression of genes coding for enzymes involved in the *de novo* ceramides synthesis pathway were observed among the studied groups (Figures 5B and S4). Interestingly, alterations in sphingomyelins (SM), which can be metabolized to ceramides through the sphingomyelinase pathway, were found (Figure S5).

### MALDI‐MSI shows heterogenous spatial distribution of intramyocellular lipids in PDAC patients

**Figure 6:**
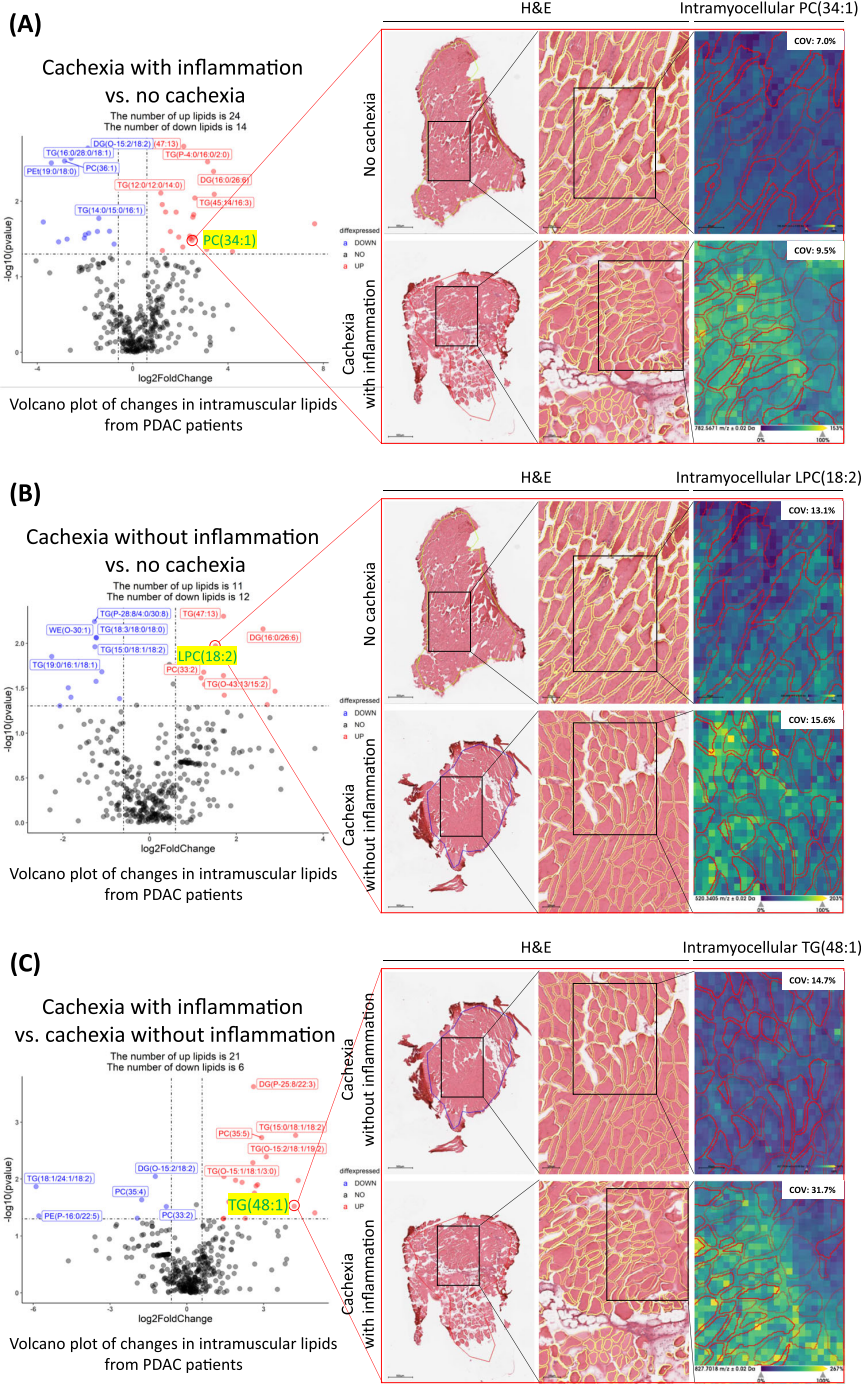
Distribution of lipid species with altered intramyocellular abundance in PDAC patients. Left panels: Volcano plots of intramyocellular lipid species identified by LC–MS/MS in the depicted patient groups. No cachexia (*n* = 10), cachexia without inflammation (*n* = 20), cachexia with inflammation (*n* = 10). Each point in the volcano graph represents a single lipid species. Red dots indicate upregulation and blue dots indicate downregulation, using a *P*‐value threshold of 0.05 (horizontal black dotted line) and a fold change of >1.5 or <−1.5 (vertical black dotted lines). Right panels: MALDI‐MSI revealed differences in the spatial distribution of PC(34:1), LPC(18:2), and TG(48:1) in the designated patient groups. Representative histological and molecular images are depicted. COV, coefficient of variation; H&E, haematoxylin and eosin staining.

Volcano plots based on LC–MS/MS‐based lipidomics analyses revealed several clear alterations in the abundance of specific intramyocellular lipid species between the different study groups (Figure 6A–C, left). To visualize the spatial distribution of these intramyocellular lipid species in cancer cachexia, untargeted MALDI‐MSI on muscle sections of 3 randomly selected patients from each study group was performed. Although the ceramide species that were identified by LC–MS/MS could not be detected using MALDI‐MSI, we were able to detect several of the altered intramyocellular glycerolipids and glycerophospholipids. These altered lipids showed a clearly heterogenous distribution in muscle. For example, PC(34:1), which was more abundant in the muscle of cachectic patients with inflammation as compared with non‐cachectic patients (Figure 6A, left), was clearly detectable in muscle sections of the former, but not the latter patients (Figure 6A, right). Similarly, a higher intensity of LPC(18:2) was found in the myofibers of cachectic patients without inflammation as compared with patients without cachexia (Figure 6B). Furthermore, consistent with the volcano plot (Figure 6C, left), cachectic patients with inflammation had more abundant intramyocellular TG(48:1) compared with cachectic patients without inflammation (Figure 6C, right). The corresponding MS/MS spectra that confirm the molecular identities of these lipids are shown in Supplemental Figure S6. For the large majority of lipid species that were detected, there was a clear heterogeneity in their distribution across the muscle. For example, PC(34:1), which was more abundant in patients with cachexia and inflammation, showed a more uniform distribution over the muscle [coefficient of variation (COV): 9.5%, Figure 6A], as compared with TG(48:1) (COV: 31.7%, Figure 6C) which was more abundant in cachectic patients without inflammation, with some regions exhibiting very low signal intensities. Heterogeneous distribution patterns of additional altered intramyocellular lipid species PC(35:5), PC(35:4), and PC(54:3) are also shown in Figure S7.

### Certain altered intramyocellular lipid species in cachectic patients versus non‐cachectic patients correlate with specific cachexia features

**Figure 7:**
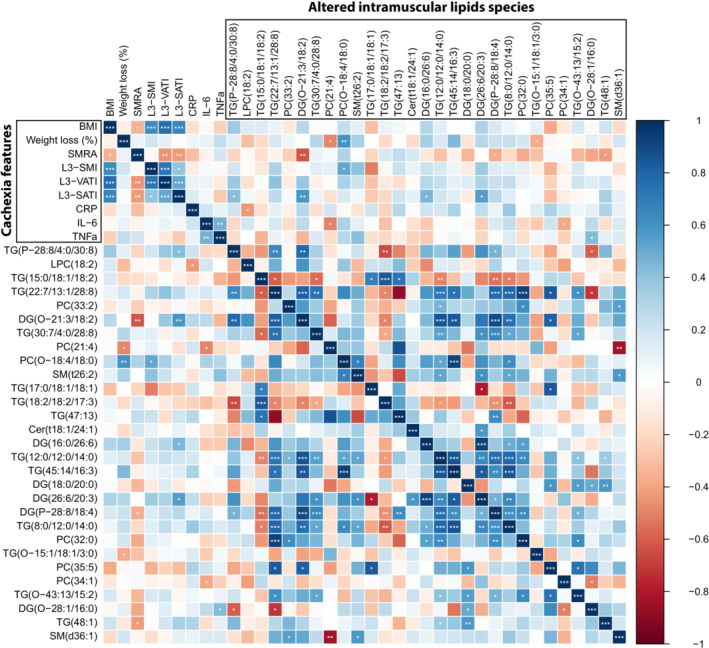
Correlation matrix of altered intramyocellular lipid species as identified by LC–MS/MS and cachexia features. The peak area of altered intramyocellular lipid species was log base 10 transformed. Blue indicates positive correlations and red indicates inverse correlations. BMI, body mass index; L3‐SMI, L3‐muscle index; SMRA, skeletal muscle radiation attenuation. Significant correlation coefficients are signified by asterisks (*P < 0.05, **P < 0.01, ***P < 0.001).

Next, we performed Spearman correlation analysis to identify potential relationships between altered intramyocellular lipid species in cachectic patients and defining features of cancer cachexia such as BMI, weight loss (%), SMRA, and the mRNA level of pro‐inflammatory cytokines in skeletal muscle. Intriguingly, several altered intramyocellular lipids such as DG(O‐21:3/18:2) and DG(P‐28:8/18:4) were negatively correlated with SMRA (*r*_*s*_ = −0.63, *P* = 0.003; *r*_*s*_ = −0.37, *P* = 0.024, respectively) (Figure 7). Similarly, intramyocellular PC(21:4) and TG(O‐15:1/18:1/3:0) were negatively correlated with weight loss (%) (*r*_*s*_ = −0.48, *P* = 0.042; *r*_*s*_ = −0.37, *P* = 0.038, respectively). In contrast, intramyocellular PC(O‐18:4/18:0) was positively correlated with weight loss (%) (*r*_*s*_ = 0.59, *P* = 0.042). PC(34:1), which abundance differed between patients with cachexia plus inflammation and non‐cachectic patients, was negatively correlated with skeletal muscle IL‐6 mRNA expression (*r*_*s*_ = −0.38, *P* = 0.048). Additional correlations between altered intramyocellular lipid species and features of cancer cachexia are presented in Figure 7.

## Discussion

In the present work, abdominal skeletal muscle tissue from PDAC patients was examined for intramyocellular alterations associated with cancer cachexia. For the first time, we utilized LC–MS/MS based‐lipidomics combined with MALDI‐MSI to explore *in situ* lipid alterations in skeletal muscle of PDAC patients with cachexia. Our results demonstrate that intramyocellular lipid accumulation in patients with cachexia is characterized by a higher relative abundance of glycerophospholipids and a lower relative abundance of glycerolipids compared with non‐cachectic patients. Furthermore, intramyocellular lipid content increased with both local inflammation (IL‐6) and systemic inflammation (CRP) in PDAC patients with or without cachexia. MALDI‐MSI revealed a heterogeneous distribution of intramyocellular lipid species with differential abundance in PDAC patients with cachexia versus those without cachexia.

It is well known that systemic inflammation plays a key role in the pathophysiology of cancer cachexia.^1^ Numerous studies have reported that elevated systemic inflammation signified by increased levels of IL‐6, TNF‐α, and CRP contributes to cancer cachexia and is associated with poor survival.^31^, ^32^ In this study, a cut‐off value of 10 mg/L for systemic CRP was used to subdivide cachectic patients into groups with or without inflammation. Fat infiltration in the skeletal muscle, also known as myosteatosis, increases with aging, obesity, and diabetes, and is associated with decreased muscle quality and function and increased mortality in patients.^33^, ^34^ Myosteatosis has long been recognized as a strong risk factor for cancer patients' survival that is closely associated with systemic inflammation. For example, a study in patients with unresectable pancreatic cancer or distal cholangiocarcinoma has shown that myosteatosis is associated with markers of systemic inflammation such as white blood cell count, CRP, and neutrophil‐lymphocyte ratio.^35^ In the present study, a significantly lower muscle radiation attenuation reflecting myosteatosis was found in cachectic patients with systemic inflammation as compared with patients without inflammation and non‐cachectic controls. This was in line with the higher lipid content in their muscle biopsies. Furthermore, skeletal muscle IL‐6 expression was correlated with intramyocellular lipid content, suggesting that both local and systemic inflammation may contribute to ectopic fat accumulation in cachexia.

Although increases in intramyocellular lipid droplet content have been reported in cachectic cancer patients and were shown to be associated with weight loss,^18^ little is known about the nature of the lipids that accumulate in skeletal muscle during cancer cachexia. As lipids and lipid metabolism are crucial for maintaining cell membrane structure and for conducting cellular signalling, aberrant accumulation of specific intramyocellular lipids such as diacylglycerols, sphingolipids, and fatty acids can result in cellular dysfunction, lipotoxicity, and cell death in skeletal muscle.^36^ In view of this, we performed lipidomics analysis on muscle fibres of rectus abdominis muscle from PDAC patients to specifically investigate potential alterations in intramyocellular lipid species during cachexia development. Our results show that the muscle of cachectic patients has a higher relative abundance of glycerophospholipids and a lower relative abundance of glycerolipids. In line with this finding, Xu and colleagues recently showed that the content of glycerophospholipids was significantly higher in a model of intramuscular fat infiltration.^37^ In the same study, pathway enrichment analysis revealed significant changes in lipid metabolism‐related pathways such as the glycerolipid pathway and glycerophospholipid pathway. Furthermore, Yang and colleagues revealed a distinct metabolic profile including amino acids, glycerolipids, and glycerophospholipids of cachectic patients by performing serum and urine metabolomics analysis in cancer patients.^38^ Based on the results above, altered intramyocellular glycerolipids and glycerophospholipids classes may play a role in skeletal muscle wasting of cachectic cancer patients. However, further studies are needed to investigate the functional role of intramyocellular glycerolipids and glycerophospholipids in cachexia‐related muscle aberrations.

Sphingolipids comprise a family of bioactive lipids that are structural components of biomembranes and play signalling roles in many aspects of cell biology. A study by Morigny and colleagues reported increases in plasma sphingolipids including sphingomyelins, ceramides, hexosyl‐ceramides, and lactosyl‐ceramides in mouse models of cachexia and cachectic cancer patients.^14^ More recently, alterations in circulating plasma ceramide levels were confirmed in cachectic cancer patients, and the ratio of C18‐ceramide to C24‐ceramide (C18:C24) was suggested to be a potential sexually dimorphic biomarker of pancreatic cancer‐induced cachexia.^39^ However, as we did not find alterations of intramyocellular ceramides in cachectic patients, changes in plasma ceramides may not be reflected at the level of the muscle.

Lipid distribution in human skeletal muscle using MSI has been reported in the context of Duchenne muscular dystrophy and obesity.^40^, ^41^ In cancer cachexia, MALDI‐MSI was recently used to investigate amino acid alterations in skeletal muscle tissue of cachectic mice.^19^ To date, however, no study has reported on the intramyocellular lipid distribution by using MALDI‐MSI in human cancer cachexia. Our results reveal distinct lipid distributions in the muscle of cachectic cancer patients. Of note, among the altered intramyocellular lipid species, some glycerolipids and glycerophospholipids could be visualized by MALDI‐MSI, but ceramides were not detected. This might be explained by low abundance of intramyocellular ceramides and/or inefficient ionization under the experimental MALDI conditions.

While our work provides multiple lines of evidence for significant lipid alterations in skeletal muscle during cancer cachexia, certain limitations of our study should be acknowledged. First, given the small sample size and the absence of a healthy control group, the results should be considered preliminary. In light of the efficacy of neoadjuvant chemotherapy in diminishing inflammation, the observed difference in intramyocellular lipid content between groups could potentially arise from the implementation of neoadjuvant therapy. A more comprehensive cohort study is warranted to elucidate the impact of neoadjuvant chemotherapy on intramyovellular lipid accumulation in the context of cancer cachexia. Second, although a positive correlation between intramyocellular lipid content and systemic as well as local inflammation was observed, it is unclear if the inflammation leads to the lipid alterations or vice versa. Moreover, the relative contribution of systemic versus local inflammation to the differences in intramyocellular lipid accumulation should be further explored. Additionally, the source of the altered intramyocellular lipids during cancer cachexia remains unknown, and paired muscle‐plasma lipidomics analyses will be important in the future. In this study, we were not able to investigate why altered intramyocellular lipids differed from one region to another. Application of multimodal imaging analyses combining e.g. MALDI‐MSI techniques with spatial transcriptomics/proteomics approaches or immunofluorescent stainings are required to explore this. Higher spatial resolutions may be needed to get more insight into the processes involved. The spatial resolution of our MALDI‐MSI instrument was set at 20*20 μm^2^, limiting its capacity to elucidate subcellular distribution of intramyocellular lipids. A high spatial resolution MSI approach such as ToF‐SIMS should be encouraged to provide more detail in this context. Finally, while we could visualize intramyocellular lipid distributions and their differences in cancer patients with and without cachexia using MALDI‐MSI, the functional importance of these differences in relation to muscle wasting remains to be investigated.

In conclusion, this study demonstrates novel differences in intramyocellular lipid accumulation in PDAC patients in relation to their inflammatory and cachectic status, with a higher relative abundance of glycerophospholipids and a lower relative abundance of glycerolipids in cachectic subjects. Increased intramyocellular lipid accumulation was associated with markers of local and systemic inflammation. These data set the stage for follow‐up studies that, we believe, should be directed towards identifying the functional impact of cachexia‐associated intramyocellular lipid alterations and the influence of both local and systemic inflammatory events on their accumulation.

## Conflict of interest

The authors declare no current or potential conflicts of interest.

## Supporting information


**Figure S1.** Correlation matrix of study variables. Positive correlations are shown in blue and inverse correlations are displayed in red. BMI: body mass index; SMRA: skeletal muscle radiation attenuation; L3‐SMI: L3‐muscle index; L3‐VATI: L3‐visceral adipose tissue index; L3‐SATI: L3‐subcutaneous adipose tissue index; CRP: C‐reactive protein. Significant correlation coefficients are signified by asterisks (* *p* < 0.05, ** *p* < 0.01, ***p < 0.001).

**Figure S2.** Morphological characteristics of abdominal skeletal muscle from PDAC patients. (A) Representative images of Haematoxylin and eosin (H&E) stained abdominal skeletal muscle tissue from patients without cachexia (top panel), cachectic patients with inflammation (bottom panel), and cachectic patients without inflammation (middle panel). (B) Relative frequency distribution of muscle fibre sizes in abdominal skeletal muscle biopsies. (C) Mean minimal Feret's diameter of muscle fibres in abdominal skeletal muscle tissue of the indicated patient groups. No cachexia (*n* = 4); cachectic without inflammation (*n* = 7); cachectic with inflammation (n = 7). For statistical analysis, 2‐way ANOVA followed by Tukey multiple comparsons test and the Kruskal‐Wallis test followed by Dunn's multiple comparisons test were used for figure S2B and figure S2C, respectively. Significant differences among the groups are signified by asterisks (*cachexia with inflammation vs. No cachexia, + cachexia with inflammation vs. cachexia without inflammation, * + *p* < 0.05, *** *p* < 0.001).

**Figure S3.** Expression of genes related to inflammation and muscle atrophy in skeletal muscle tissue from PDAC patients is related to intramyocellular lipid content. mRNA expression of TNF‐α (A, upper panel) and IL‐6 (B, upper panel) in skeletal muscle from non‐cachectic patients and cachexia patients. mRNA expression of TNF‐α (A, lower panel) and IL‐6 (B, lower panel) in skeletal muscle from non‐cachectic patients and cachexia patients with or without inflammation. Correlation between intramyocellular lipid content (%) and the mRNA expression of inflammatory genes TNF‐α (C), IL‐6 (D) in skeletal muscle, and systemic CRP level (E). The line reflects the median; the hinges of the boxes are drawn at the 25th and 75th percentile. The dots, squares and triangles in figure A‐B reflect the outliers as defined by the Tukey method. For statistical analysis, the Mann–Whitney U test was used for analysis of two groups; the Kruskal‐Wallis test followed by Dunn's multiple comparisons test was used for analysis of multiple groups. For correlation analysis, Spearman's rank correlation coefficient (*r*_*s*_) was used for the relationship between variables. The level of significance is indicated in the respective plots.

**Figure S4.** mRNA expression of genes coding for enzymes controlling de novo ceramides synthesis in abdominal skeletal muscle of cachectic PDAC patients (*n* = 30) versus non‐cachectic PDAC patients (*n* = 10). For statistical analysis, the Mann–Whitney U test was used for two groups. The line reflects the median; the hinges of the boxes are drawn at the 25th and 75th percentile. The dots and squares reflect the outliers as defined by the Tukey method. Significant differences among the groups are signified by asterisks (* *p* < 0.05).

**Figure S5.** Relative level of intramyocellular sphingomyelins in PDAC patients. Comparison of intramyocellular sphingomyelins in skeletal muscle from PDAC patients. Unpaired student's *t*‐test was conducted. (*, **, and *** indicate differences from the control group (no cachexia). Data are presented as mean + SEM, ** *p* < 0.01).

**Figure S6.** Representative MALDI‐MSI MS/MS spectra. MS/MS Spectra of [PC(34:1) + Na]^+^ (A), [LPC(18:2) + H]^+^ (B), and [TG(48:1) + Na]^+^ (C) in positive mode.

**Figure S7.** Distribution of additional lipid species with altered intramyocellular abundance in PDAC patients. MALDI‐MSI revealed differences in the spatial distribution of PC(35:5), PC(35:4), and PC(54:3) in the designated patient groups. No cachexia (*n* = 3), cachexia without inflammation (*n* = 3), cachexia with inflammation (*n* =3). Representative histological and molecular images are depicted. H&E: haematoxylin and eosin staining.

**Table S1.** Basic characteristics of the first consecutive 21 patients.

**Table S2.** Basic characteristics of patients for MALDI‐MSI analysis.

**Table S3.** Primer sequences for RT‐qPCR.

**Table S4.** Basic characteristics of patients used for myofibers size measurement.

**Table S5.** Basic characteristics of the 40 patients included for lipidomics.

**Table S6.** Supporting Information.
