# Supplementary figures and images for: Inflammation‐associated intramyocellular lipid alterations in human pancreatic cancer cachexia
Source: J Cachexia Sarcopenia Muscle. 2024 May 9;15(4):1283–97. doi: 10.1002/jcsm.13474 (PMC11294036; doi:10.1002/jcsm.13474)

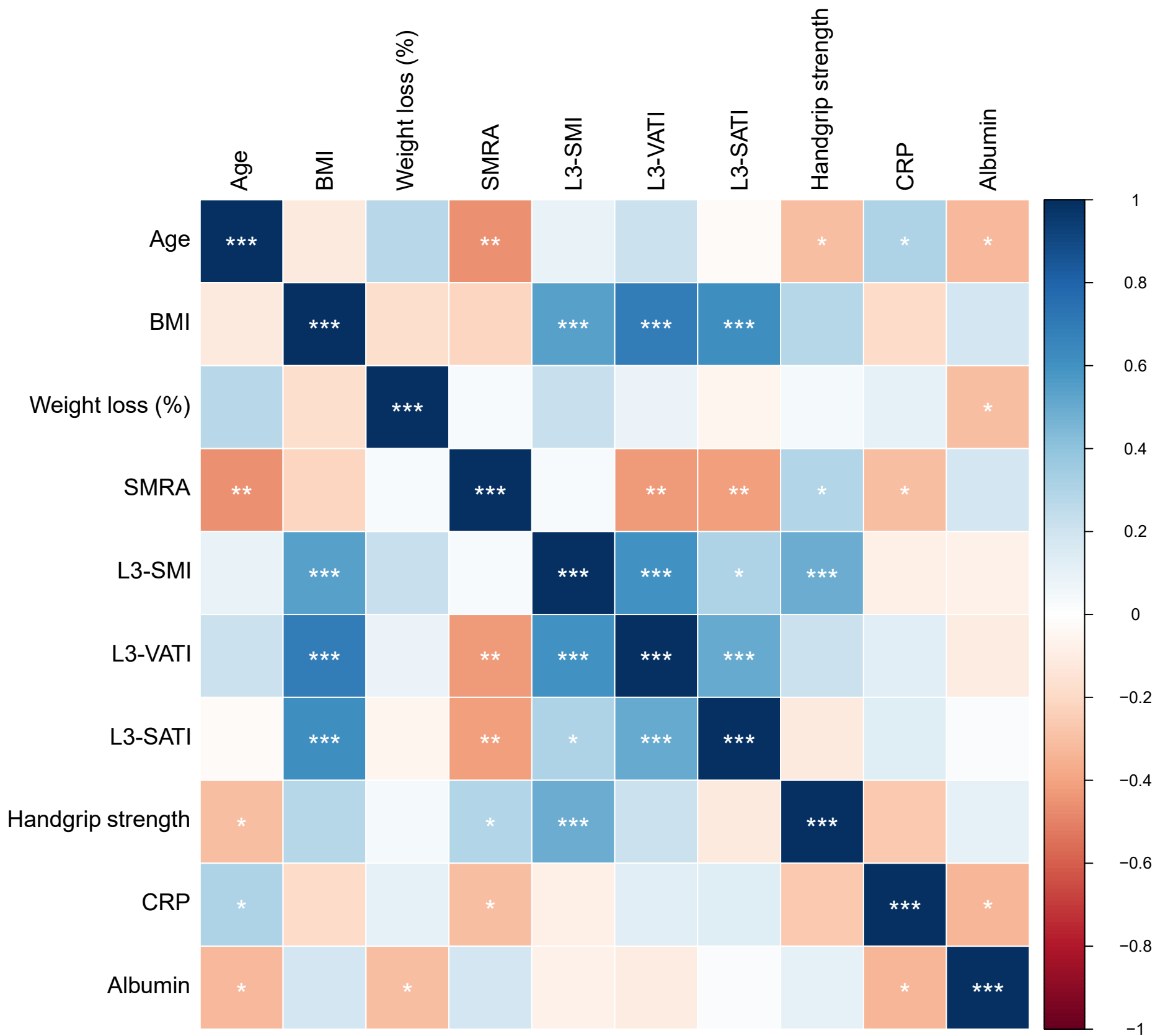

Supplement: Supplementary file 1 — Figure S1. Correlation matrix of study variables. Positive correlations are shown in blue and inverse correlations are displayed in red. BMI: body mass index; SMRA: skeletal muscle radiation attenuation; L3‐SMI: L3‐muscle index; L3‐VATI: L3‐visceral adipose tissue index; L3‐SATI: L3‐subcutaneous adipose tissue index; CRP: C‐reactive protein. Significant correlation coefficients are signified by asterisks (* p < 0.05, ** p < 0.01, ***p < 0.001). [file JCSM-15-1283-s001.pdf]

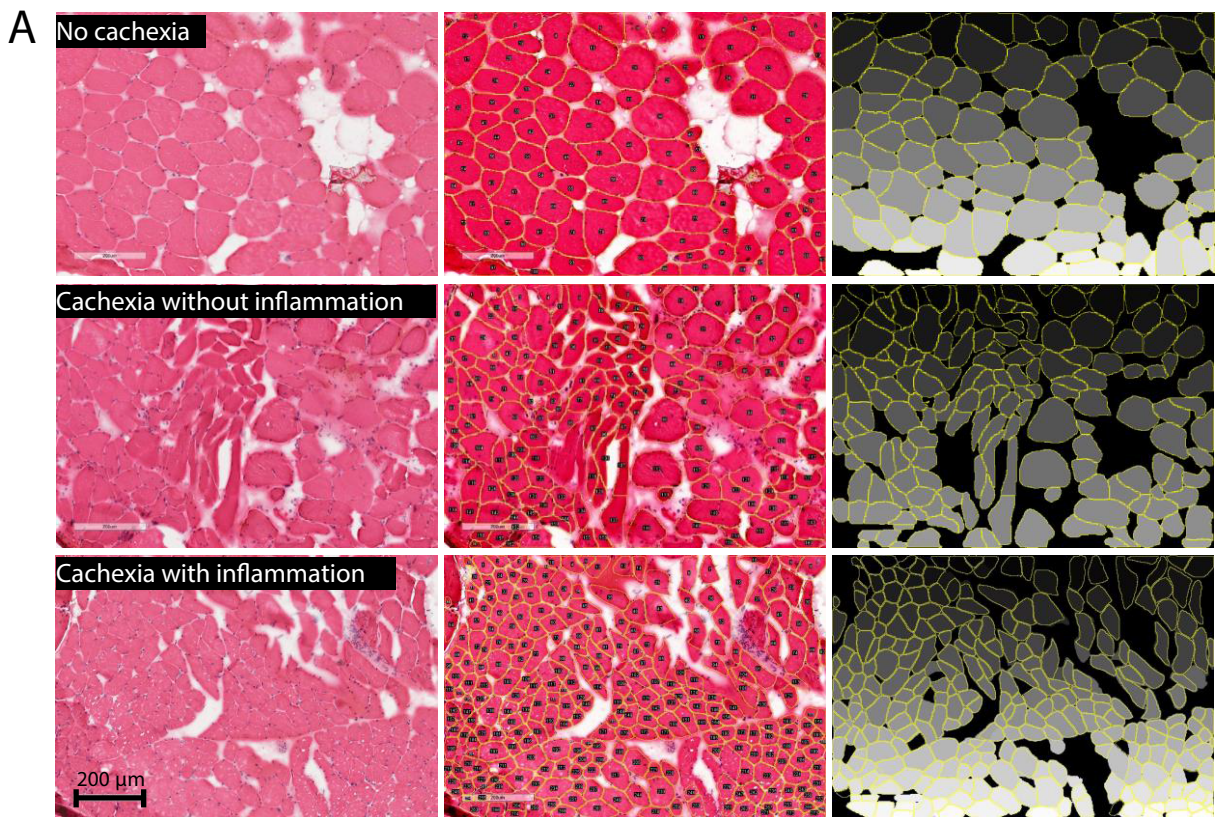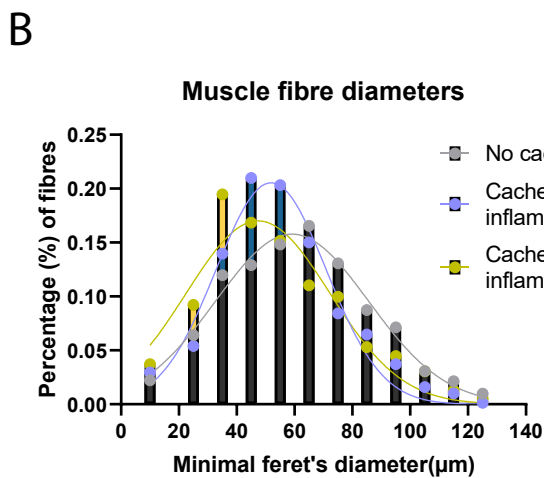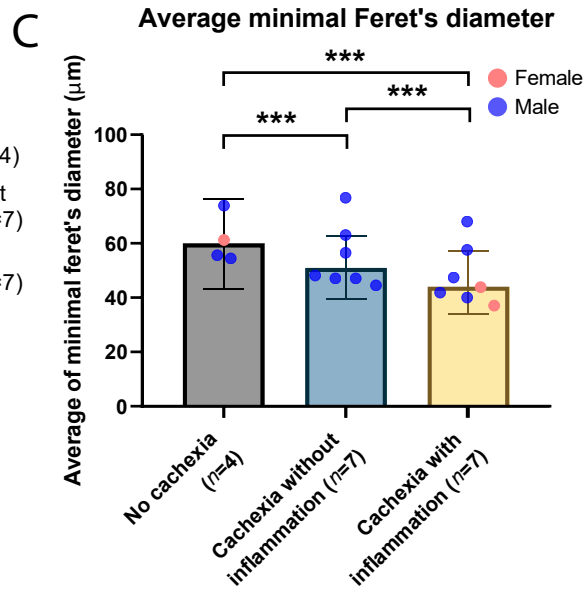

Supplement: Supplementary file 2 — Figure S2. Morphological characteristics of abdominal skeletal muscle from PDAC patients. (A) Representative images of Haematoxylin and eosin (H&E) stained abdominal skeletal muscle tissue from patients without cachexia (top panel), cachectic patients with inflammation (bottom panel), and cachectic patients without inflammation (middle panel). (B) Relative frequency distribution of muscle fibre sizes in abdominal skeletal muscle biopsies. (C) Mean minimal Feret's diameter of muscle fibres in abdominal skeletal muscle tissue of the indicated patient groups. No cachexia (n = 4); cachectic without inflammation (n = 7); cachectic with inflammation (n = 7). For statistical analysis, 2‐way ANOVA followed by Tukey multiple comparsons test and the Kruskal‐Wallis test followed by Dunn's multiple comparisons test were used for figure S2B and figure S2C, respectively. Significant differences among the groups are signified by asterisks (*cachexia with inflammation vs. No cachexia, + cachexia with inflammation vs. cachexia without inflammation, * + p < 0.05, *** p < 0.001). [file JCSM-15-1283-s006.pdf]

**A**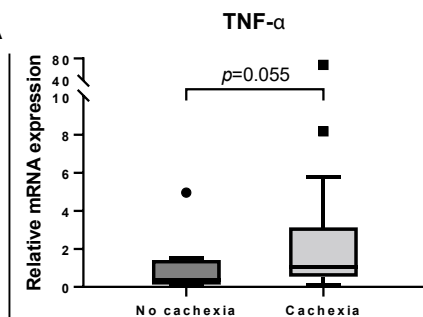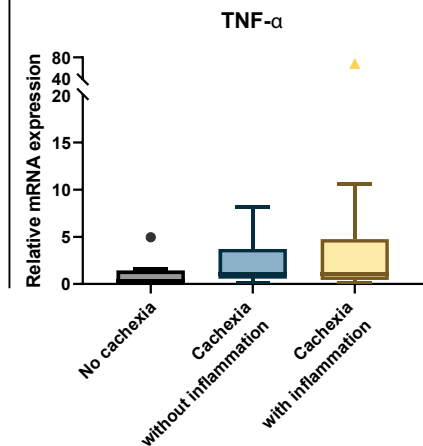**B**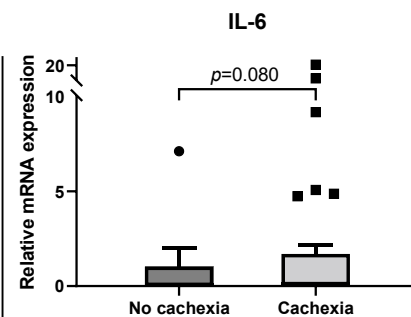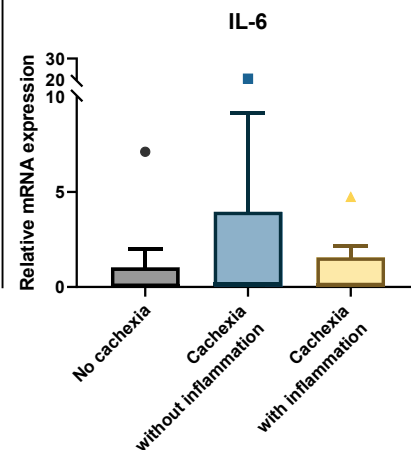**C**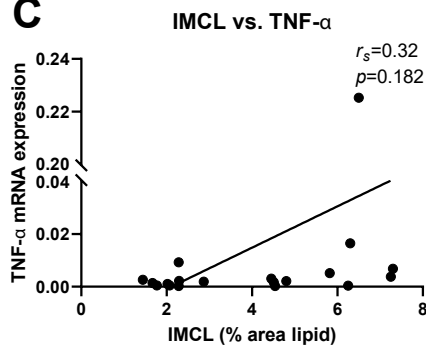**D**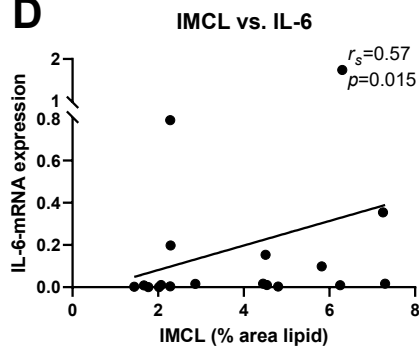**E**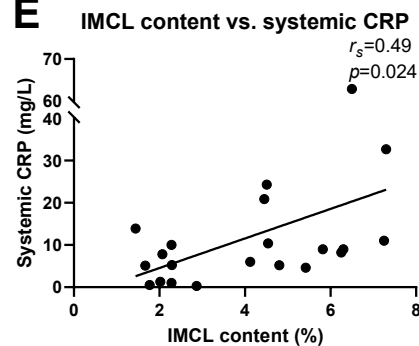

Supplement: Supplementary file 3 — Figure S3. Expression of genes related to inflammation and muscle atrophy in skeletal muscle tissue from PDAC patients is related to intramyocellular lipid content. mRNA expression of TNF‐α (A, upper panel) and IL‐6 (B, upper panel) in skeletal muscle from non‐cachectic patients and cachexia patients. mRNA expression of TNF‐α (A, lower panel) and IL‐6 (B, lower panel) in skeletal muscle from non‐cachectic patients and cachexia patients with or without inflammation. Correlation between intramyocellular lipid content (%) and the mRNA expression of inflammatory genes TNF‐α (C), IL‐6 (D) in skeletal muscle, and systemic CRP level (E). The line reflects the median; the hinges of the boxes are drawn at the 25th and 75th percentile. The dots, squares and triangles in figure A‐B reflect the outliers as defined by the Tukey method. For statistical analysis, the Mann–Whitney U test was used for analysis of two groups; the Kruskal‐Wallis test followed by Dunn's multiple comparisons test was used for analysis of multiple groups. For correlation analysis, Spearman's rank correlation coefficient (r s ) was used for the relationship between variables. The level of significance is indicated in the respective plots. [file JCSM-15-1283-s004.pdf]

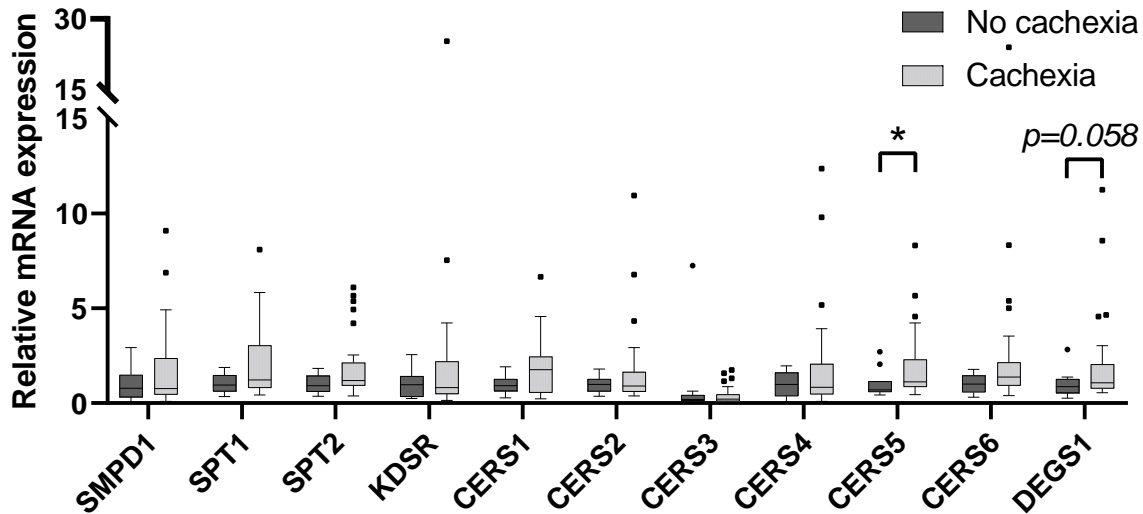

Supplement: Supplementary file 4 — Figure S4. mRNA expression of genes coding for enzymes controlling de novo ceramides synthesis in abdominal skeletal muscle of cachectic PDAC patients (n = 30) versus non‐cachectic PDAC patients (n = 10). For statistical analysis, the Mann–Whitney U test was used for two groups. The line reflects the median; the hinges of the boxes are drawn at the 25th and 75th percentile. The dots and squares reflect the outliers as defined by the Tukey method. Significant differences among the groups are signified by asterisks (* p < 0.05). [file JCSM-15-1283-s005.pdf]

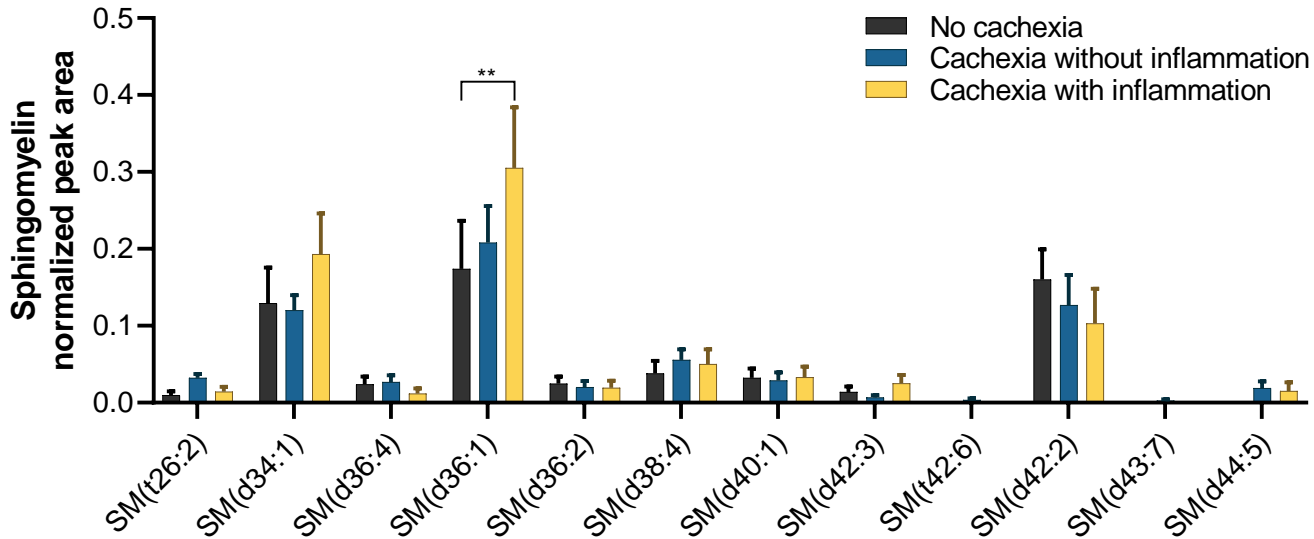

Supplement: Supplementary file 5 — Figure S5. Relative level of intramyocellular sphingomyelins in PDAC patients. Comparison of intramyocellular sphingomyelins in skeletal muscle from PDAC patients. Unpaired student's t‐test was conducted. (*, **, and *** indicate differences from the control group (no cachexia). Data are presented as mean + SEM, ** p < 0.01). [file JCSM-15-1283-s007.pdf]

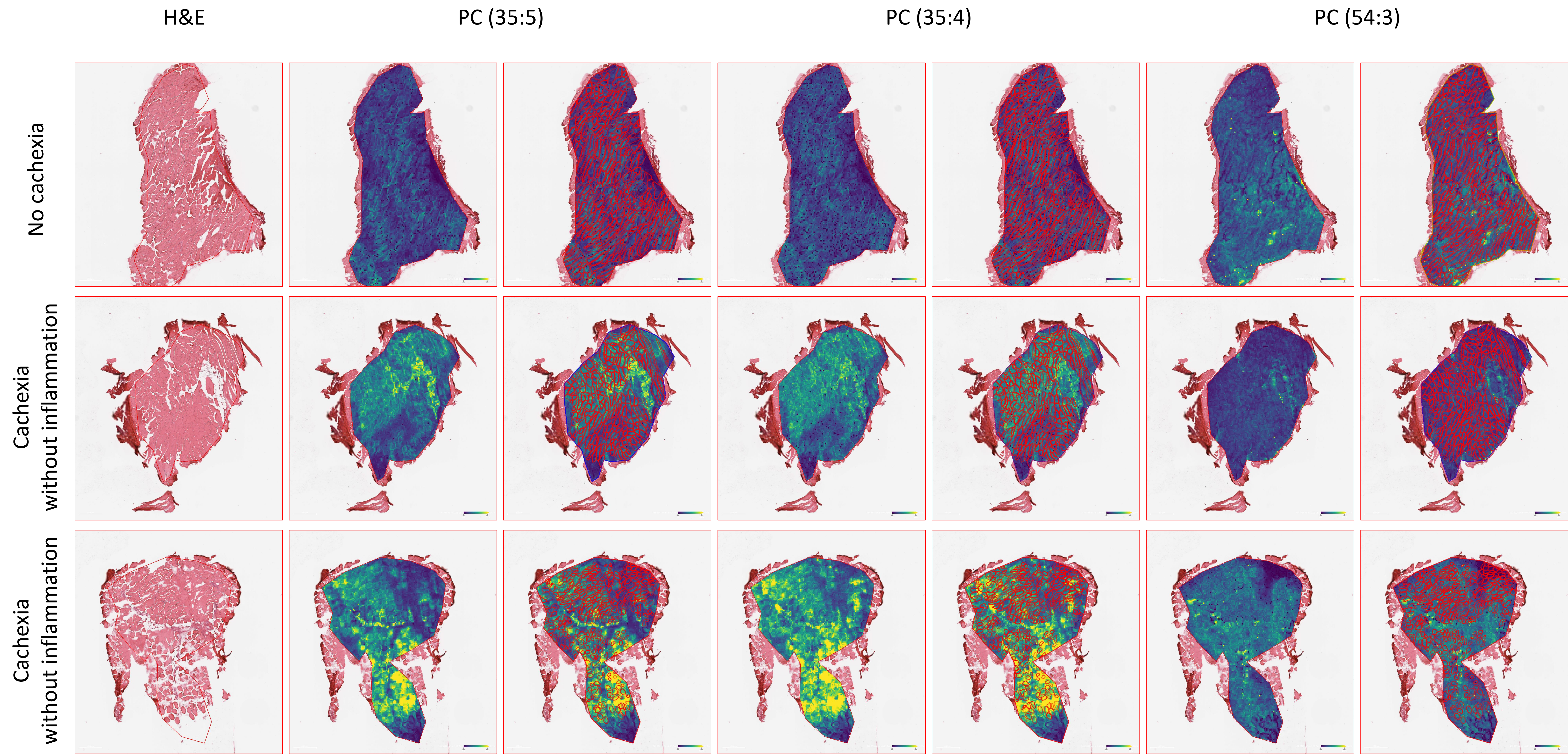

Supplement: Supplementary file 7 — Figure S7. Distribution of additional lipid species with altered intramyocellular abundance in PDAC patients. MALDI‐MSI revealed differences in the spatial distribution of PC(35:5), PC(35:4), and PC(54:3) in the designated patient groups. No cachexia (n = 3), cachexia without inflammation (n = 3), cachexia with inflammation (n =3). Representative histological and molecular images are depicted. H&E: haematoxylin and eosin staining. [file JCSM-15-1283-s008.pdf]
