# Supplementary material for: Inflammation‐associated intramyocellular lipid alterations in human pancreatic cancer cachexia
Source: J Cachexia Sarcopenia Muscle. 2024 May 9;15(4):1283–97. doi: 10.1002/jcsm.13474 (PMC11294036; doi:10.1002/jcsm.13474)
Supplement: Supplementary file 6 — Figure S6. Representative MALDI‐MSI MS/MS spectra. MS/MS Spectra of [PC(34:1) + Na]+ (A), [LPC(18:2) + H]+ (B), and [TG(48:1) + Na]+ (C) in positive mode. [file JCSM-15-1283-s003.pdf]

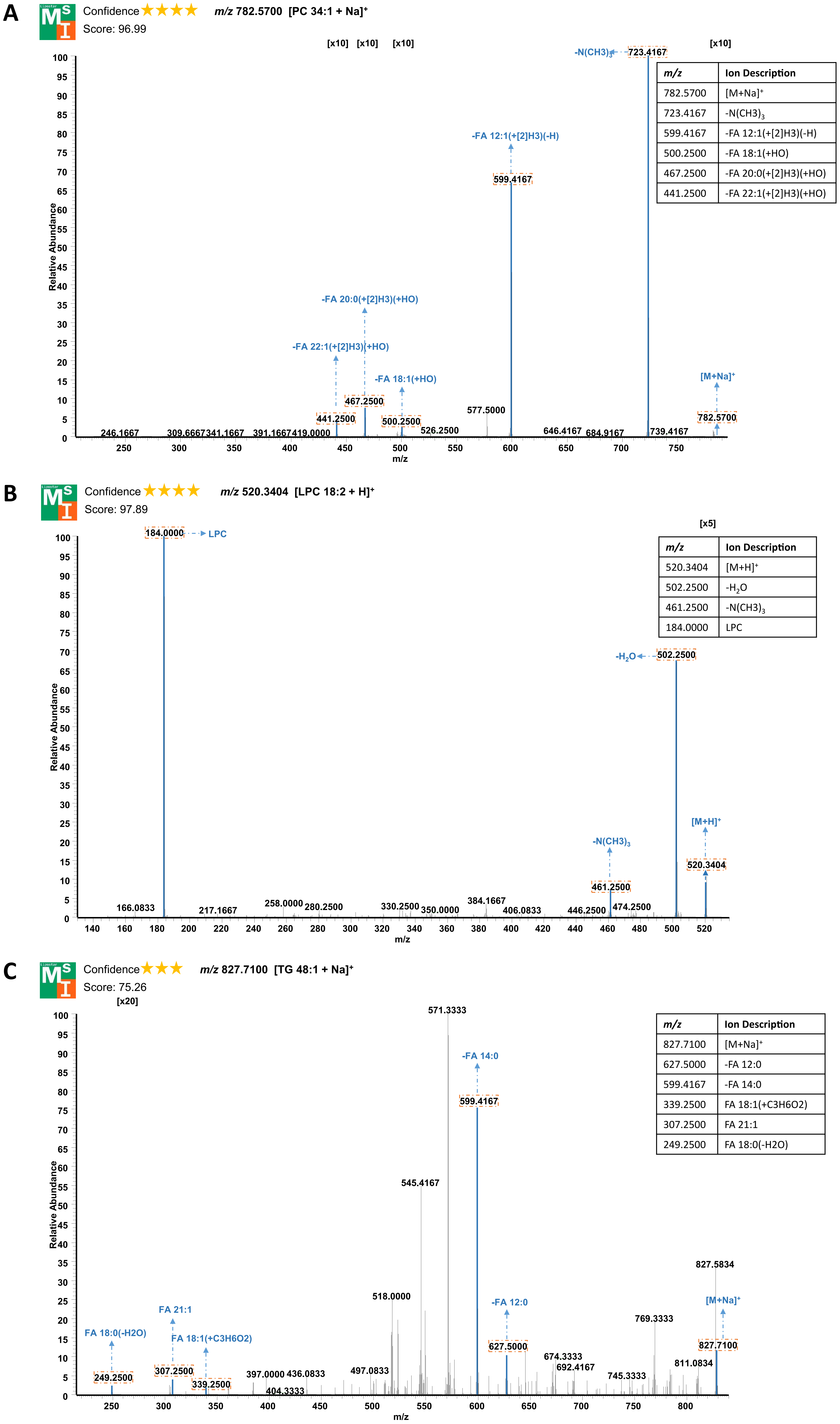
A
Confidence
m/z 782.5700 [PC 34:1 + Na]+
Score: 96.99
Relative Abundance
m/z
-N(CH3)3
-FA 12:1(+[2]H3)(-H)
-FA 20:0(+[2]H3)(+HO)
-FA 22:1(+[2]H3)(+HO)
-FA 18:1(+HO)
[M+Na]+
m/z | Ion Description
782.5700 | [M+Na]+
723.4167 | -N(CH3)3
599.4167 | -FA 12:1(+[2]H3)(-H)
500.2500 | -FA 18:1(+HO)
467.2500 | -FA 20:0(+[2]H3)(+HO)
441.2500 | -FA 22:1(+[2]H3)(+HO)
B
Confidence
m/z 520.3404 [LPC 18:2 + H]+
Score: 97.89
Relative Abundance
m/z
LPC
-H2O
-N(CH3)3
[M+H]+
m/z | Ion Description
520.3404 | [M+H]+
502.2500 | -H2O
461.2500 | -N(CH3)3
184.0000 | LPC
C
Confidence
m/z 827.7100 [TG 48:1 + Na]+
Score: 75.26
Relative Abundance
m/z
-FA 14:0
-FA 12:0
FA 21:1
FA 18:0(-H2O)
FA 18:1(+C3H6O2)
[M+Na]+
m/z | Ion Description
827.7100 | [M+Na]+
627.5000 | -FA 12:0
599.4167 | -FA 14:0
339.2500 | FA 18:1(+C3H6O2)
307.2500 | FA 21:1
249.2500 | FA 18:0(-H2O)
