# Supplementary material for: Inflammation‐associated intramyocellular lipid alterations in human pancreatic cancer cachexia
Source: J Cachexia Sarcopenia Muscle. 2024 May 9;15(4):1283–97. doi: 10.1002/jcsm.13474 (PMC11294036; doi:10.1002/jcsm.13474)
Supplement: Supplementary file 8 — Table S1. Basic characteristics of the first consecutive 21 patients. [file JCSM-15-1283-s013.docx]

**Supplementary Table S1**: Basic characteristics of the first consecutive 21 patients

|  | **Overall** | **No cachexia** | **Cachexia** | **Cachexia** | ***p*** |
| --- | --- | --- | --- | --- | --- |
|  |  |  | **without inflammation** | **with inflammation** |  |
| *n* | 21 | 6 | 9 | 6 |  |
| Age (years) | 71.0 (67.0, 75.0) | 61.0 (58.8, 71.5) | 72.0 (69.0, 75.0) | 72.0 (68.2, 75.8) | 0.373 |
| Sex = F/M (%) | 7/14 (33.3/66.7) | 3/3 (50.0/50.0) | 2/7 (22.2/77.8) | 2/4 (33.3/66.7) | 0.642 |
| BMI (kg/m^2^) | 23.0 (22.2, 25.4) | 24.0 (22.4, 25.3) | 22.8 (22.2, 24.8) | 23.4 (21.8, 25.8) | 0.938 |
| Weight Loss (%) | 8.8 (5.0, 14.5) | 2.3 (1.6, 3.3) | 13.9 (10.4, 15.7)^†^ | 8.7 (8.0, 13.1)^†^ | 0.001 |
| Handgrip strength (kg) | 30.0 (24.0, 40.0) | 34.0 (30.0, 44.0) | 27.0 (26.0, 33.0) | 27.0 (22.0, 41.8) | 0.643 |
| SMRA (HU) | 31.2 (27.2, 38.8) | 37.8 (34.7, 42.9) | 31.2 (30.3, 38.5) | 24.2 (21.0, 26.7) | 0.110 |
| L3-SMI (cm^2^/m^2^) | 42.6 (35.2, 46.7) | 39.7 (31.9, 44.9) | 45.1 (38.2, 50.6) | 36.5 (34.0, 47.5) | 0.249 |
| Male | 45.8 (43.1, 50.6) | 45.7 (44.2, 45.8) | 46.7 (44.8, 52.0) | 44.4 (37.2, 51.8) | 0.702 |
| Female | 34.8 (29.0, 36.0) | 30.3 (28.5, 33.5) | 36.7 (36.0, 37.5) | 31.2 (29.5, 33.0) | 0.297 |
| L3-VATI (cm^2^/m^2^) | 41.0 (24.7, 62.1) | 22.6 (17.7, 39.8) | 58.6 (27.5, 86.5) | 44.7 (31.9, 58.7) | 0.201 |
| Male | 56.7 (35.8, 81.2) | 44.9 (28.0, 49.8) | 65.3 (45.7, 87.0) | 55.2 (42.7, 78.9) | 0.293 |
| Female | 20.5 (14.9, 26.8) | 20.5 (18.6, 22.6) | 10.2 (8.7, 11.6) | 35.0 (31.9, 38.0) | 0.069 |
| L3-SATI (cm^2^/m^2^) | 44.0 (38.8, 49.8) | 43.9 (33.5, 49.7) | 44.0 (38.8, 48.9) | 44.9 (42.3, 54.8) | 0.860 |
| Male | 45.1 (39.0, 50.1) | 39.8 (35.0, 45.0) | 44.0 (39.8, 49.3) | 52.0 (40.1, 64.5) | 0.671 |
| Female | 43.6 (36.6, 46.8) | 48.0 (39.7, 67.6) | 38.0 (34.3, 41.8) | 42.8 (42.3, 43.2) | 0.555 |
| CRP (mg/L) | 8.2 (5.1, 11.0) | 6.4 (2.2, 9.4) | 5.3 (4.6, 8.2) | 22.6 (13.5, 30.6)^†‡^ | 0.003 |
| Albumin (g/dL) | 3.8 (3.1, 4.3) | 4.3 (4.3, 4.4) | 3.4 (2.9, 4.4) | 3.2 (3.0, 3.6)^†^ | 0.049 |
| CRP/albumin ratio | 2.0 (1.1, 3.5) | 1.2 (0.3, 1.8) | 2.0 (1.0, 2.1) | 6.8 (4.8, 8.5)^†‡^ | 0.002 |
| Cancer Stage (%) |  |  |  |  | 0.472 |
| IA | 2 (9.5) | 0 (0.0) | 1 (11.1) | 1 (16.7) |  |
| IIA | 3 (14.3) | 0 (0.0) | 2 (22.2) | 1 (16.7) |  |
| IIB | 11 (52.4) | 5 (83.3) | 2 (22.2) | 4 (66.7) |  |
| III | 1 (4.8) | 0 (0.0) | 1 (11.1) | 0 (0.0) |  |
| IV^&^ | 2 (9.5) | 0 (0.0) | 2 (22.2) | 0 (0.0) |  |
| Neoadjuvant chemotherapy (%) |  |  |  |  | 0.138 |
| No | 15 (71.4) | 6 (100.0) | 4 (44.4) | 5 (83.3) |  |
| Yes | 1 (4.8) | 0 (0.0) | 1 (11.1) | 0 (0.0) |  |
| Unknown | 5 (23.8) | 0 (0.0) | 4 (44.4) | 1 (16.7) |  |

The data are presented as median + IQR. Groups were compared using the Kruskal–Wallis test followed by Dunn’s post-testing. † Significant difference in comparison to the no cachexia group. ‡ Significant difference in comparison to the cachexia without inflammation group. BMI: body mass index; HU: Hounsfield unit; SMRA: skeletal muscle radiation attenuation; L3-SMI: L3-muscle index; L3-VATI: L3-visceral adipose tissue index; L3-SATI: L3-subcutaneous adipose tissue index; CRP: C-reactive protein. &: patients underwent exploratory surgery, no resection.
