# Supplementary material for: Inflammation‐associated intramyocellular lipid alterations in human pancreatic cancer cachexia
Source: J Cachexia Sarcopenia Muscle. 2024 May 9;15(4):1283–97. doi: 10.1002/jcsm.13474 (PMC11294036; doi:10.1002/jcsm.13474)
Supplement: Supplementary file 9 — Table S2. Basic characteristics of patients for MALDI‐MSI analysis. [file JCSM-15-1283-s011.docx]

**Supplementary Table S2**: Basic characteristics of patients for MALDI-MSI analysis

|  | **Overall** | **No cachexia** | **Cachexia** | **Cachexia** | ***p*** |
| --- | --- | --- | --- | --- | --- |
|  |  |  | **without inflammation** | **with inflammation** |  |
| *n* | 9 | 3 | 3 | 3 |  |
| Age (years) | 60.0 (56.0, 71.0) | 56.0 (55.0, 58.5) | 59.0 (55.5, 59.5) | 75.0 (73.0, 76.0) | 0.066 |
| Sex = F/M (%) | 3/6 (33.3/66.7) | 2/1 (66.7/33.3) | 0/3 (0.0/100.0) | 1/2 (33.3/66.7) | 0.679 |
| BMI (kg/m^2^) | 21.9 (21.4, 25.4) | 21.9 (21.1, 23.6) | 26.8 (24.4, 27.0) | 21.4 (21.1, 22.0) | 0.236 |
| Weight Loss (%) | 7.7 (3.2, 8.6) | 2.3 (1.8, 2.8) | 19.8 (13.2, 20.5) | 8.4 (8.1, 8.5) | 0.061 |
| Handgrip strength (kg) | 32.0 (26.0, 46.0) | 38.0 (32.0, 42.0) | 46.0 (39.0, 48.0) | 22.0 (21.0, 27.0) | 0.118 |
| SMRA (HU) | 36.2 (33.1, 44.2) | 44.2 (44.1, 44.6) | 35.2 (34.2, 39.7) | 23.0 (20.6, 29.6) | 0.094 |
| L3-SMI (cm^2^/m^2^) | 40.8 (34.8, 44.0) | 32.8 (29.8, 37.7) | 49.7 (45.2, 51.8) | 39.0 (36.9, 41.5) | 0.148 |
| Male | 43.3 (41.2, 48.3) | 42.6 (42.6, 42.6) | 49.7 (45.2, 51.8) | 41.5 (40.2, 42.8) | 0.538 |
| Female | 32.8 (29.8, 33.8) | 29.8 (28.2, 31.3) | NA (NA, NA) | 34.8 (34.8, 34.8) | 0.221 |
| L3-VATI (cm^2^/m^2^) | 25.8 (16.8, 48.1) | 16.8 (13.9, 17.8) | 75.4 (43.9, 82.0) | 41.0 (33.4, 44.5) | 0.193 |
| Male | 37.0 (15.7, 68.6) | 11.0 (11.0, 11.0) | 75.4 (43.9, 82.0) | 37.0 (31.4, 42.5) | 0.304 |
| Female | 18.7 (17.8, 29.9) | 17.8 (17.3, 18.2) | NA (NA, NA) | 41.0 (41.0, 41.0) | 0.221 |
| L3-SATI (cm^2^/m^2^) | 41.9 (31.4, 44.4) | 31.4 (30.8, 37.9) | 47.7 (39.4, 55.2) | 41.9 (41.1, 42.5) | 0.491 |
| Male | 41.7 (33.4, 46.5) | 30.1 (30.1, 30.1) | 47.7 (39.4, 55.2) | 41.7 (41.0, 42.4) | 0.304 |
| Female | 41.9 (36.6, 43.1) | 37.9 (34.6, 41.1) | NA (NA, NA) | 41.9 (41.9, 41.9) | 1.000 |
| CRP (mg/L) | 5.0 (0.8, 47.0) | 1.3 (0.9, 26.8) | 0.8 (0.7, 2.9) | 47.0 (38.5, 55.0) | 0.177 |
| Albumin (g/dL) | 4.4 (4.3, 4.6) | 4.3 (4.3, 4.4) | 4.5 (4.4, 4.7) | 4.0 (3.5, 4.3) | 0.390 |
| CRP/albumin ratio | 1.0 (0.2, 10.0) | 0.3 (0.2, 6.2) | 0.2 (0.2, 0.6) | 10.0 (8.8, 15.2) | 0.161 |
| Cancer Stage (%) |  |  |  |  | 1.000 |
| IIB | 2 (22.2) | 1 (33.3) | 0 (0.0) | 1 (33.3) |  |
| IV^&^ | 5 (55.6) | 1 (33.3) | 2 (66.7) | 2 (66.7) |  |
| Unknown | 2 (22.2) | 1 (33.3) | 1 (33.3) | 0 (0.0) |  |
| Neoadjuvant  chemotherapy (No/Yes (%)) | 6/3 (66.7/33.3) | 2/1 (66.7/33.3) | 1/2 (33.3/66.7) | 3/0 (100.0/0.0) | 0.679 |

The data are presented as median + IQR. Groups were compared using the Kruskal–Wallis test followed by Dunn’s post-testing. † Significant difference in comparison to the no cachexia group. ‡ Significant difference in comparison to the cachexia without inflammation group. BMI: body mass index; HU: Hounsfield unit; SMRA: skeletal muscle radiation attenuation; L3-SMI: L3-muscle index; L3-VATI: L3-visceral adipose tissue index; L3-SATI: L3-subcutaneous adipose tissue index; CRP: C-reactive protein. &: Patients underwent exploratory surgery, no resection.
