# Supplementary material for: Inflammation‐associated intramyocellular lipid alterations in human pancreatic cancer cachexia
Source: J Cachexia Sarcopenia Muscle. 2024 May 9;15(4):1283–97. doi: 10.1002/jcsm.13474 (PMC11294036; doi:10.1002/jcsm.13474)
Supplement: Supplementary file 10 — Table S3. Primer sequences for RT‐qPCR. [file JCSM-15-1283-s009.docx]

**Supplementary Table S3**: Primer sequences for RT-qPCR

| **Gene** | **Forward (5’-3’)** | **Reverse (5’-3’)** |
| --- | --- | --- |
| RPLP0 | TCTACAACCCTGAAGTGCTTGATATC | GCAGACAGACACTGGCAACATT |
| TNF-α | CTGGGCAGGTCTACTTTGGG | CTGGAGGCCCCAGTTTGAAT |
| IL-6 | TCCAGGAGCCCAGCTATGAA | GAGCAGCCCCAGGGAGAA |
| SMPD1 | CACACGGTAACCAGGATTAAGG | CCTCAGAATTGGGGGGTTCTATGC |
| SPT1 | ACATCGTTTCAGGCCCTCCA | TCCCCACGCCATACTTCTTT |
| SPT2 | TGCCAAAATTGGCGCCTT | GTACCAACCGATGACGGGAA |
| KDSR | ACATGGTGTCTCCGCTCATCA | TATGCAAAGCACCACCTGTTT |
| Cers1 | GAGCTACAGTGCCTACCTGC | TTGTGGTACCGGAAGGCGTA |
| Cers2 | CGAGAAGCCAGCTGGAGATT | AATGCTGAAGAGCAGGGACC |
| Cers3 | TGGCTGGAGTCTGCTAAGATG | TAGGCAAGATCAGCGTGCAA |
| Cers4 | CAGACCAGGAGGCAAGTGAA | AGCCATGACTCGTGGTACAG |
| Cers5 | GCCACTGGCAACATACTCCT | CGGCAATGAAACTCACGCAT |
| Cers6 | GGAACGCTGGTCCTTTGTCT | TGGTATTTAACACCCAGAGAGGA |
| DEGS1 | GAGCTGATGGCGTCGATGTA | GACCTGTGCCACGGTATTGA |
