# Supplementary material for: Inflammation‐associated intramyocellular lipid alterations in human pancreatic cancer cachexia
Source: J Cachexia Sarcopenia Muscle. 2024 May 9;15(4):1283–97. doi: 10.1002/jcsm.13474 (PMC11294036; doi:10.1002/jcsm.13474)
Supplement: Supplementary file 11 — Table S4. Basic characteristics of patients used for myofibers size measurement. [file JCSM-15-1283-s002.docx]

**Supplementary Table S4**: Basic characteristics of patients used for myofibers size measurement

|  | **Overall** | **No cachexia** | **Cachexia** | **Cachexia** | ***p*** |
| --- | --- | --- | --- | --- | --- |
|  |  |  | **without inflammation** | **with inflammation** |  |
| *n* | 18 | 4 | 7 | 7 |  |
| Age (years) | 71.5 (61.0, 75.8) | 66.0 (57.5, 75.0) | 64.0 (59.5, 75.0) | 75.0 (68.5, 76.0) | 0.419 |
| Sex = F/M (%) | 3/15 (16.7/83.3) | 1/3 (25.0/75.0) | 0/7 (0.0/100.0) | 2/5 (28.6/71.4) | 0.400 |
| BMI (kg/m^2^) | 24.9 (22.8, 27.2) | 28.0 (24.5, 31.1) | 26.8 (23.5, 27.2) | 23.0 (22.0, 25.1) | 0.257 |
| Weight Loss (%) | 8.5 (5.7, 14.9) | 2.8 (1.5, 4.0) | 15.0 (8.4, 19.1) **^†^** | 8.6 (8.1, 11.7) | 0.009 |
| Handgrip strength (kg) | 32.0 (22.2, 45.8) | 26.5 (20.8, 34.5) | 42.0 (36.0, 46.0) | 22.0 (21.0, 38.5) | 0.161 |
| SMRA (HU) | 33.4 (25.5, 38.4) | 37.5 (35.3, 39.2) | 35.2 (31.0, 38.0) | 23.4 (21.6, 26.1) | 0.046 |
| L3-SMI (cm^2^/m^2^) | 45.5 (38.5, 51.2) | 45.8 (43.8, 47.7) | 49.7 (44.2, 52.5) | 38.3 (34.3, 44.8) | 0.211 |
| Male | 45.8 (42.0, 52.4) | 45.8 (45.8, 49.5) | 49.7 (44.2, 52.5) | 39.0 (38.3, 50.5) | 0.544 |
| Female | 34.8 (31.2, 36.3) | 37.9 (37.9, 37.9) | NA (NA, NA) | 31.2 (29.5, 33.0) | 0.221 |
| L3-VATI (cm^2^/m^2^) | 51.5 (35.2, 85.3) | 49.8 (42.0, 68.1) | 75.4 (35.5, 92.9) | 48.1 (35.0, 55.2) | 0.809 |
| Male | 58.6 (46.5, 92.9) | 54.7 (49.8, 81.4) | 75.4 (35.5, 92.9) | 48.4 (48.1, 62.1) | 0.966 |
| Female | 33.2 (31.0, 37.1) | 33.2 (33.2, 33.2) | NA (NA, NA) | 35.0 (31.9, 38.0) | 1.000 |
| L3-SATI (cm^2^/m^2^) | 47.0 (39.9, 61.4) | 60.5 (47.6, 85.0) | 47.7 (35.0, 55.8) | 43.6 (41.1, 52.0) | 0.443 |
| Male | 47.7 (39.3, 60.2) | 50.2 (45.0, 60.5) | 47.7 (35.0, 55.8) | 46.2 (40.4, 57.7) | 0.827 |
| Female | 43.6 (42.8, 85.7) | 127.8 (127.8, 127.8) | NA (NA, NA) | 42.8 (42.3, 43.2) | 0.221 |
| CRP (mg/L) | 5.0 (2.0, 19.1) | 4.7 (3.8, 7.3) | 1.1 (0.9, 3.3) | 24.3 (15.9, 39.9) **^‡^** | 0.002 |
| Albumin (g/dL) | 4.1 (3.3, 4.5) | 4.3 (4.2, 4.3) | 4.5 (4.2, 4.5) | 3.3 (3.0, 3.8) | 0.063 |
| CRP/albumin ratio | 1.0 (0.5, 5.6) | 1.0 (0.8, 1.1) | 0.3 (0.2, 0.8) | 8.1 (5.0, 9.3) **^‡^** | 0.002 |
| Cancer Stage (%) |  |  |  |  | 0.553 |
| IA | 1 (5.9) | 0 (0.0) | 0 (0.0) | 1 (16.7) |  |
| IIA | 2 (11.8) | 0 (0.0) | 1 (14.3) | 1 (16.7) |  |
| IIB | 8 (47.1) | 2 (50.0) | 2 (28.6) | 4 (66.7) |  |
| IV^&^ | 2 (11.8) | 1 (25.0) | 1 (14.3) | 0 (0.0) |  |
| Unknown | 4 (23.5) | 1 (25.0) | 3 (42.9) | 0 (0.0) |  |
| Neoadjuvant chemotherapy (%) |  |  |  |  | 0.666 |
| No | 12 (66.7) | 3 (75.0) | 4 (50.0) | 5 (83.3) |  |
| Yes | 3 (16.7) | 1 (25.0) | 2 (25.0) | 0 (0.0) |  |
| Unknown | 3 (16.7) | 0 (0.0) | 2 (25.0) | 1 (16.7) |  |

The data are presented as median + IQR. Groups were compared using the Kruskal–Wallis test followed by Dunn’s post-testing. † Significant difference in comparison to the no cachexia group. ‡ Significant difference in comparison to the cachexia without inflammation group. BMI: body mass index; HU: Hounsfield unit; SMRA: skeletal muscle radiation attenuation; L3-SMI: L3-muscle index; L3-VATI: L3-visceral adipose tissue index; L3-SATI: L3-subcutaneous adipose tissue index; CRP: C-reactive protein. &: Patients underwent exploratory surgery, no resection.
