# Supplementary material for: Inflammation‐associated intramyocellular lipid alterations in human pancreatic cancer cachexia
Source: J Cachexia Sarcopenia Muscle. 2024 May 9;15(4):1283–97. doi: 10.1002/jcsm.13474 (PMC11294036; doi:10.1002/jcsm.13474)
Supplement: Supplementary file 12 — Table S5. Basic characteristics of the 40 patients included for lipidomics. [file JCSM-15-1283-s012.docx]

**Supplementary Table S5**: Basic characteristics of the 40 patients included for lipidomics

|  | **Overall** | **No cachexia** | **Cachexia** | **Cachexia** | ***p*** |
| --- | --- | --- | --- | --- | --- |
|  |  |  | **without inflammation** | **with inflammation** |  |
| *n* | 40 | 10 | 20 | 10 |  |
| Age (years) | 69.0 (59.8, 75.0) | 61.0 (56.5, 73.0) | 68.5 (59.8, 75.0) | 73.0 (68.2, 76.0) | 0.296 |
| Sex = F/M (%) | 12/28 (30.0/70.0) | 4/6 (40.0/60.0) | 4/16 (20.0/80.0) | 4/6 (40.0/60.0) | 0.434 |
| BMI (kg/m2) | 23.0 (22.1, 25.8) | 24.0 (22.3, 25.5) | 23.0 (22.1, 25.9) | 22.9 (21.6, 25.8) | 0.868 |
| Weight Loss (%) | 8.8 (4.7, 14.5) | 2.1 (0.8, 3.0) | 13.6 (10.2, 16.4) ^†^ | 8.7 (7.9, 12.8) ^†^ | <0.001 |
| Handgrip strength (kg) | 32.0 (24.8, 40.5) | 34.0 (27.0, 44.0) | 32.5 (26.0, 40.5) | 24.0 (20.5, 32.0) | 0.229 |
| SMRA (HU) | 34.0 (27.7, 39.0) | 36.3 (33.9, 42.9) | 36.2 (30.0, 39.6) | 26.1 (23.1, 30.8) | 0.042 |
| L3-IMAT (cm2) | 5.3 (4.3, 14.1) | 4.7 (3.9, 8.8) | 5.2 (4.2, 6.4) | 12.2 (7.1, 18.4) | 0.059 |
| L3-IMATI (cm2/m2) | 1.8 (1.3, 3.7) | 1.7 (1.2, 2.8) | 1.6 (1.2, 2.0) | 3.7 (2.7, 5.9) | 0.107 |
| Male | 1.6 (1.2, 2.8) | 1.4 (0.9, 2.7) | 1.6 (1.2, 1.7) | 5.0 (1.7, 5.9) | 0.488 |
| Female | 2.8 (2.0, 4.7) | 1.9 (1.7, 2.8) | 3.7 (2.9, 4.5) | 3.2 (2.8, 4.7) | 0.229 |
| L3-SMI (cm2/m2) | 41.2 (37.1, 47.5) | 40.4 (33.8, 44.9) | 43.8 (38.0, 49.9) | 39.0 (35.7, 48.9) | 0.452 |
| Male | 44.8 (40.1, 50.5) | 44.2 (41.6, 45.8) | 45.2 (40.7, 50.8) | 41.5 (38.5, 48.9) | 0.751 |
| Female | 35.0 (30.8, 38.4) | 31.5 (29.4, 33.8) | 36.7 (34.2, 39.0) | 36.8 (33.0, 42.8) | 0.309 |
| L3-VATI (cm2/m2) | 38.8 (20.2, 74.1) | 24.8 (19.1, 52.3) | 35.0 (15.2, 86.8) | 44.5 (31.8, 58.7) | 0.540 |
| Male | 51.5 (25.8, 87.8) | 49.8 (29.9, 81.8) | 62.0 (25.4, 87.8) | 48.2 (31.4, 58.7) | 0.968 |
| Female | 22.6 (16.6, 34.2) | 19.6 (18.2, 21.6) | 14.4 (11.6, 20.0) | 40.7 (37.5, 49.2) ^‡^ | 0.031 |
| L3-SATI (cm2/m2) | 44.2 (36.5, 62.5) | 46.2 (33.5, 65.6) | 42.4 (31.0, 53.0) | 44.9 (42.2, 61.3) | 0.613 |
| Male | 41.9 (30.9, 52.1) | 45.0 (32.5, 65.6) | 39.8 (30.4, 49.1) | 44.6 (41.0, 54.8) | 0.698 |
| Female | 46.8 (43.2, 74.7) | 46.2 (41.1, 57.8) | 58.0 (41.8, 82.2) | 53.0 (43.2, 72.3) | 0.926 |
| CRP (mg/L) | 5.2 (1.2, 11.7) | 4.8 (1.5, 9.4) | 2.8 (1.0, 5.2) | 27.1 (18.5, 43.4) ^†‡^ | <0.001 |
| Albumin (g/dL) | 4.0 (3.3, 4.4) | 4.3 (4.1, 4.3) | 4.0 (3.3, 4.4) | 3.5 (3.1, 4.0) | 0.137 |
| CRP/albumin ratio | 1.2 (0.3, 3.6) | 1.1 (0.3, 1.8) | 0.8 (0.3, 1.9) | 7.8 (4.8, 9.7) ^†‡^ | <0.001 |
| Cancer Stage (%) |  |  |  |  | 0.041 |
| IA | 2 (5.1) | 0 (0.0) | 1 (5.3) | 1 (10.0) |  |
| IB | 1 (2.6) | 0 (0.0) | 0 (0.0) | 1 (10.0) |  |
| IIA | 5 (12.8) | 1 (10.0) | 3 (15.8) | 1 (10.0) |  |
| IIB | 15 (38.5) | 5 (50.0) | 3 (15.8) | 7 (70.0) |  |
| III | 3 (7.7) | 1 (10.0) | 2 (10.5) | 0 (0.0) |  |
| IV^&^ | 5 (12.8) | 0 (0.0) | 5 (26.3) | 0 (0.0) |  |
| Unknown | 8 (20.5) | 3 (30.0) | 5 (26.3) | 0 (0.0) |  |
| Neoadjuvant chemotherapy (%) |  |  |  |  | 0.064 |
| No | 22 (55.0) | 7 (70.0) | 7 (35.0) | 8 (80.0) |  |
| Yes | 10 (25.0) | 3 (30.0) | 6 (30.0) | 1 (10.0) |  |
| Unknown | 8 (20.0) | 0 (0.0) | 7 (35.0) | 1 (10.0) |  |

The data are presented as median + IQR. Groups were compared using the Kruskal–Wallis test followed by Dunn’s post-testing. † Significant difference in comparison to the no cachexia group. ‡ Significant difference in comparison to the cachexia without inflammation group. BMI: body mass index; HU: Hounsfield unit; SMRA: skeletal muscle radiation attenuation; L3-SMI: L3-muscle index; L3-VATI: L3-visceral adipose tissue index; L3-SATI: L3-subcutaneous adipose tissue index; CRP: C-reactive protein. &: Patients underwent exploratory surgery, no resection.
